# Safeguarding pollinators requires specific habitat prescriptions and substantially more land area than suggested by current policy

**DOI:** 10.1038/s41598-022-26872-x

**Published:** 2023-03-21

**Authors:** Alana Pindar, Nigel E. Raine

**Affiliations:** 1 School of Science and Techonology, Cape Breton University, Sydney, NS B1P 6L2 Canada; 2 School of Environmental Sciences, University of Guelph, Guelph, ON N1G 2W1 Canada

**Keywords:** Conservation biology, Ecosystem services

## Abstract

Habitat loss and fragmentation are major drivers of global pollinator declines, yet even after recent unprecedented periods of anthropogenic land-use intensification the amount of habitat needed to support insect pollinators remains unknown. Here we use comprehensive pan trap bee survey datasets from Ontario, Canada, to determine which habitat types are needed and at what spatial scales to support wild bee communities. Safeguarding wild bee communities in a Canadian landscape requires 11.6–16.7% land-cover from a diverse range of habitats (~ 2.6–3.7 times current policy guidelines) to provide targeted habitat prescriptions for different functional guilds over a variety of spatial scales, irrespective of whether conservation aims are enhancing bee species richness or abundance. Sensitive and declining habitats, like tallgrass woodlands and wetlands, were important predictors of bee biodiversity. Conservation strategies that under-estimate the extent of habitat, spatial scale and specific habitat needs of functional guilds are unlikely to protect bee communities and the essential pollination services they provide to both crops and wild plants.

## Introduction

Human-induced land-use changes are driving unprecedented, widespread and increasing global biodiversity losses^1,2^. These alarming declines in biodiversity result in the degradation of many essential ecosystem services and functions^3,4^, including pollination. Indeed, wild bees and the pollination services they provide to crops and wild plants are experiencing global declines in response to intensive anthropogenic landscape changes, climate change, parasites and diseases, competition from invasive species, and rising agrochemical usage^5–7^.

The Sustainable Development Agenda set globally agreed goals to end poverty, protect the planet, and ensure peace and prosperity for all by 2030^8^. However, less than a decade from this deadline little apparent progress has been made towards many of these key targets, including the need to ‘ensure the conservation, restoration and sustainable use of terrestrial and inland freshwater ecosystems and their services’ (Goal 15.1)^8^ including pollination services. Efforts to slow, or even reverse global pollinator declines have led many countries to initiate conservation strategies in agricultural areas^9–11^, urban environments^12^, and other sensitive lands to mitigate the loss of vital pollinators and the ecosystem services they provide^5,7^. Selection and implementation of specific conservation strategies will strongly depend on conservation priorities and may differ substantially if the goal is to: (1) enhance pollination by pollinators visiting particular crops^13,14^, (2) maintain wider pollinator biodiversity^13^ or (3) specifically target the recovery of pollinator species-at-risk^15^.

**Figure 1:**
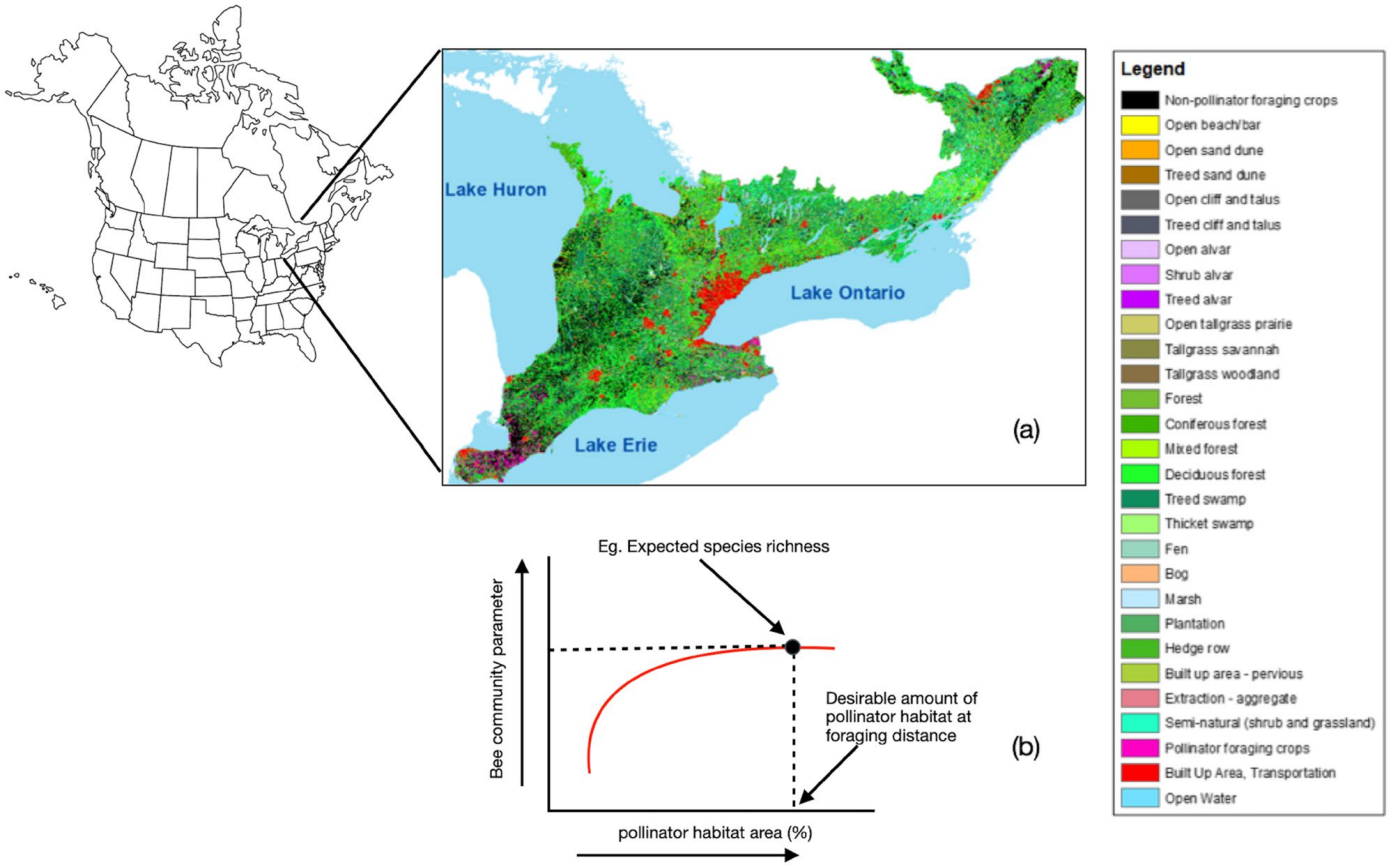
(**a**) Landscape gradient across Southern Ontario, Canada (Ecoregions 5E and 6E) a North American landscape. Red (urban areas), black (intensive wind pollinated crops), and light blue (open water areas) reflect areas that provide little or no pollinator habitat. Pink represents intensive agricultural crops that provide pollinator foraging habitat, while light- to darker-green colours represent a gradient of natural and semi-natural habitats; (**b**) The expected relationship between extent of pollinator habitat and the bee species richness supported in the landscape. Initial increases in the amount of pollinator habitat in a landscape are associated with a steep increase in bee species richness. However, the slope of this red line become shallower with additional increases in the extent of pollinator habitat, until it reaches an asymptote—signifying the optimal landscape composition to support maximal bee species richness (marked with black dotted lines). Map was produced using SOLRIS v.2.1^60^ and ACI data^61^ in ArcGIS v. 10.6.x (https://desktop.arcgis.com/en/system-requirements/10.6/arcgis-desktop-system-requirements.htm).

In 2016, the government of Ontario, Canada, mandated provincial policy to “restore, enhance and protect one million acres of pollinator habitat”^16^, which represents about 4.5% of the land area of Southern Ontario (mixed wood plain ecozone) where most of province’s rich agricultural lands are situated. As part of Ontario’s provincial Pollinator Health Action Plan^16^, the Ministry of Natural Resources and Forestry (MNRF) was tasked with the action of ‘assessing land cover in natural habitats, and in agricultural and urban landscapes in southern Ontario to identify and map probable pollinator habitat’. The creation of the pollinator habitat (PHaB) mapping layer revealed that Southern Ontario has over ~ 2.7 million hectares of pollinator habitat (approximately 20% of the total area: Fig. 1a), representing 24 different habitat types (see Ontario Land Classes in Tables S4, S4). However, creating the PHaB mapping layer also revealed that there has been a net loss of nearly 10,000 hectares of pollinator habitat in Ontario over the 10-year period 2002–2012^17^.

Whilst one million acres (or 404,686 hectares) of pollinator habitat sounds like an impressively large area, there is no evidence to suggest that this much pollinator-suitable habitat will be sufficient to conserve healthy communities of wild pollinators and the essential ecosystem services they provide for agricultural production^18–20^ and native plant communities^21^ in a landscape. Although studies have shown the importance of specific habitat types (such as semi-natural and natural habitat, urban, and consistent foraging crops) for particular bee species at various spatial scales and over quality gradients in a landscape^10,22–26^, there still remain significant fundamental knowledge gaps on basic relationships between most pollinator species and habitat types^27^. The species-area relationship is one of the most studied patterns in ecology, with many studies using the relationship as a tool to better understand biological diversity and habitats^28,29^. Fundamentally, the slope of the species-area curve predicts the number of species found within an area. However, and quite surprisingly, there is not yet any clear understanding of how much of each specific habitat type is required to support a pollinator community, or indeed over what spatial scale such habitats are needed. This lack of information not only severely limits the ability to make and implement evidence-based recommendations to support pollinators at local or landscape scales, but also jeopardizes the chances of meeting globally agreed Sustainable Development Goals^30^ and the success of government policy, such as those outlined by the province of Ontario.

While bee species richness and abundance are tightly linked to availability and quality of floral and nesting resources, these associations do not necessarily predict how much of a specific habitat is needed by any species^31,32^. In most ecosystems the provision of any additional suitable habitat will increase pollinator abundance and diversity^31,32^. Here, we use an extensive dataset of bees including ~ 66,000 observations from 361 species, 86% of the species recorded from Ontario, Canada, from published surveys conducted over a 12-year period. Specifically we aim to explore the relationship between the maximum number of bee species representing commonly used functional guilds: (1) solitary ground nesters, (2) social ground nesters, (3) cavity nesters, (4) bumblebees or *Bombus* spp. (except subgenus *Psithyrus*), and (5) cleptoparasites and social parasites (including *Bombus* subgenus *Psithyrus*) and the amount of specific habitat types they require at three spatial scales in a landscape. Our main objective in this work is to provide evidence-based information on the amount of habitat types needed to support pollinators at local and landscape scales to aid in reaching sustainable conservation measures of these important species.

## Results

### How much habitat is needed?

**Figure 2:**
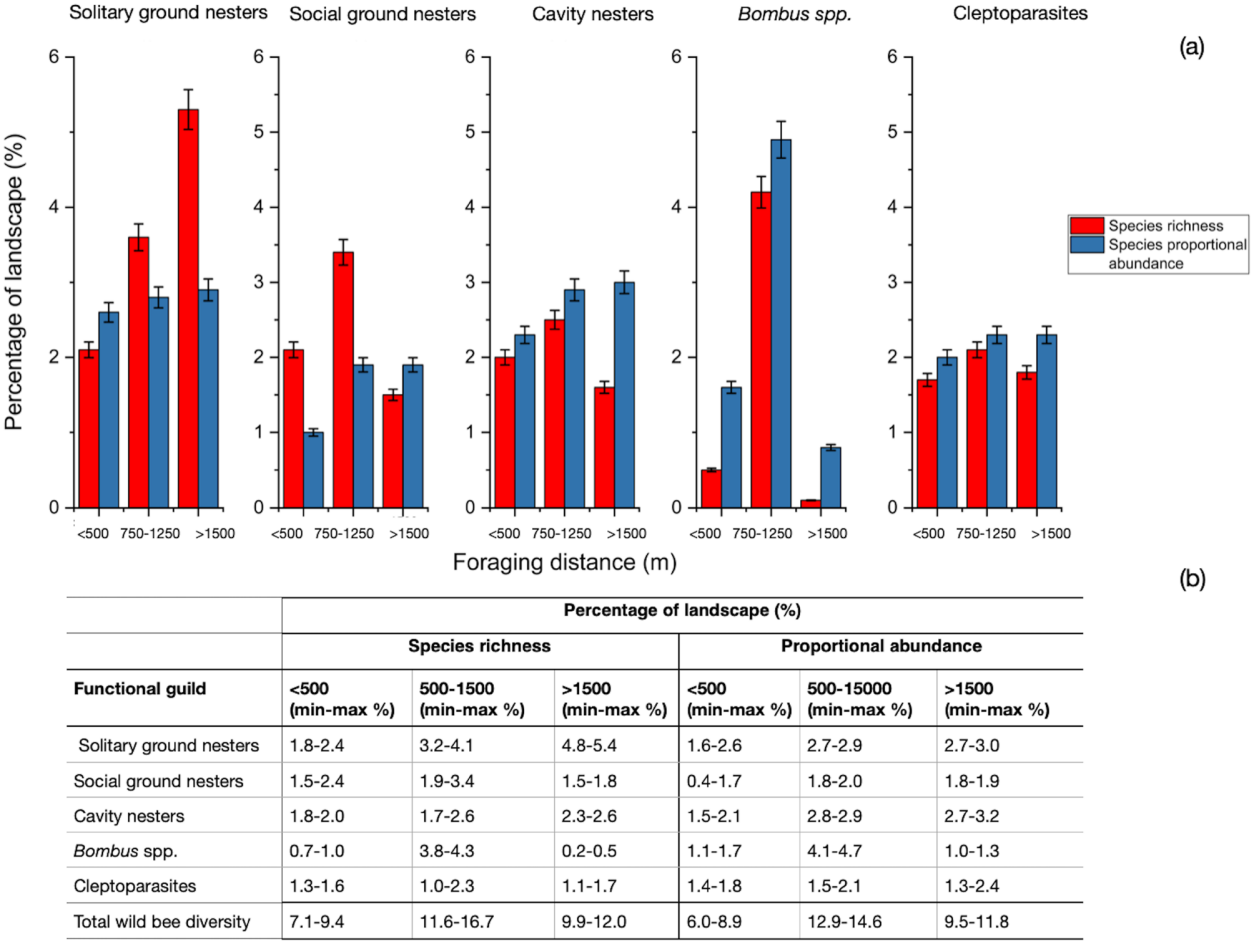
(**a**) The mean amount of habitats; (**b**) the maximum and minimum amounts of habitat within a landscape to maintain the species richness (red-) and proportional abundance (blue columns) of five functional bee guilds: solitary ground nesters, social ground nesters, cavity nesters, bumblebees (*Bombus* spp. excluding subgenus *Psithryus*), and cleptoparasitic species (including *Bombus* subg. *Psithryus*) expected community parameters at each foraging category (< 500 m, between 500 and 1500 m, and > 1500 m).

Our results suggest that different amounts of habitat are required for maintaining the highest species richness and abundance of wild bee functional guilds at different spatial scales (Fig. 2a). Specifically, we found all functional guilds, other than solitary ground nesters, showed a preference for habitat at foraging distances between 750 and 1250 m over more localized (< 500 m) and more dispersed scales (> 1500 m) for maintaining species richness (social ground nesters: $\overline{x}$= 3.4% ± 0.17; cavity nesters: $\overline{x}$= 2.5% ± 0.13; *Bombus spp.*: $\overline{x}$= 4.2% ± 0.21; cleptoparasites: $\overline{x}$= 2.1% ± 0.10) (Fig. 2a). In contrast, more habitat occupying a greater percentage of the landscape at larger spatial scales would be needed to support a higher richness of solitary ground-nesting species (> 1500 m: $\overline{x}$= 5.3% ± 0.27) (Fig. 2a). Only bumblebees (*Bombus* spp.) showed the same significant trend for maintaining both the highest abundance and species-richness from habitat needs between 750 and 1250 m (Fig. 2a). We found no differences in the amount of habitat needed to support the abundance of solitary ground nesters among spatial categories (< 500 m: $\overline{x}$= 2.6% ± 0.10; 750 m-1250 m: $\overline{x}$= 2.8% ± 0.14; > 1500 m: $\overline{x}$= 2.9% ± 0.14). The abundance of social ground nesters, cavity nesters, and cleptopasites was not significantly different when considering the amount of habitat required between 750 and 1250 m and > 1500 m, however all exhibited increased abundance from localized (< 500 m) to larger scales of habitat extent (750–1250 m) (Fig. 2a,b).

**Figure 3:**
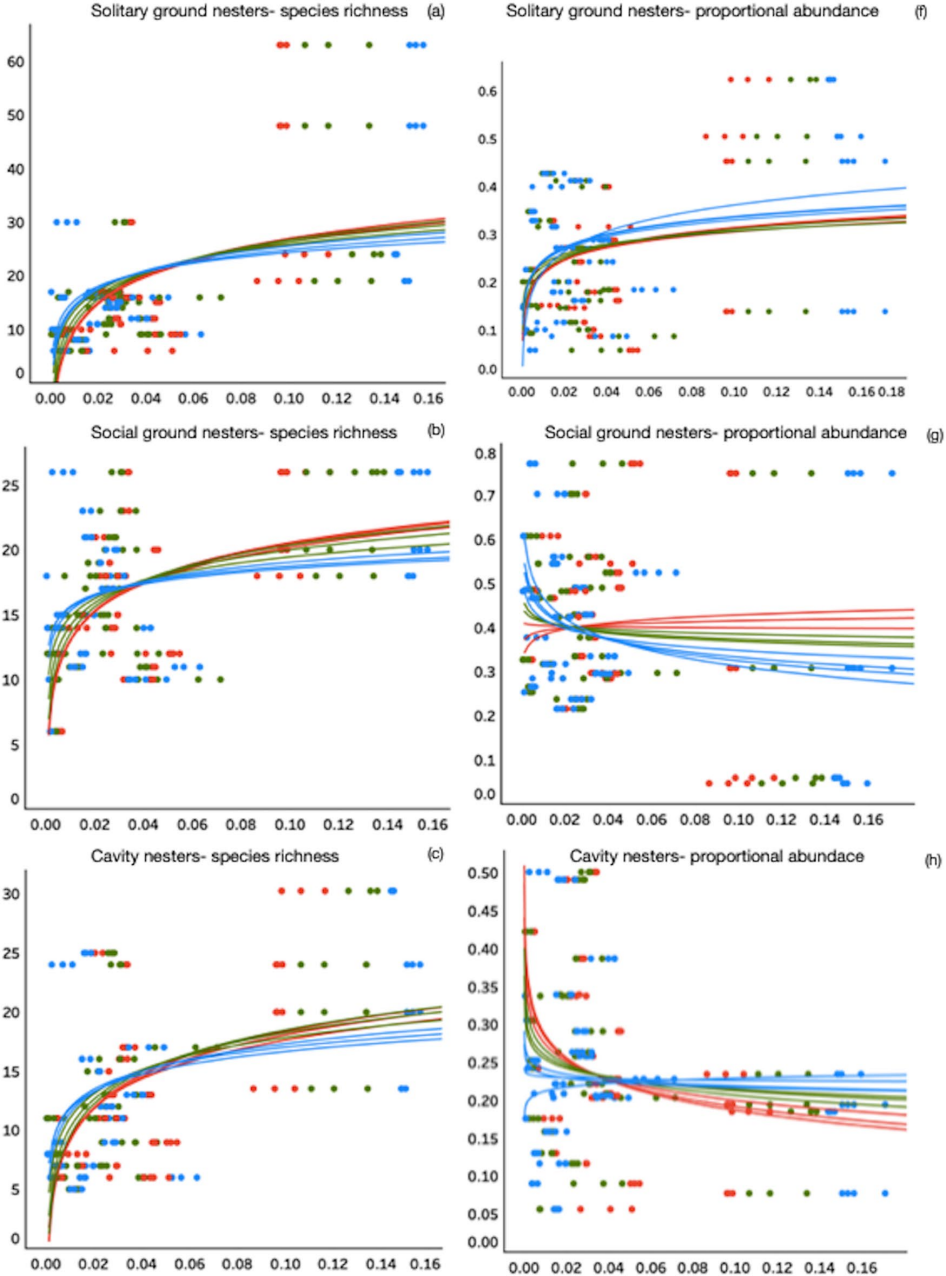

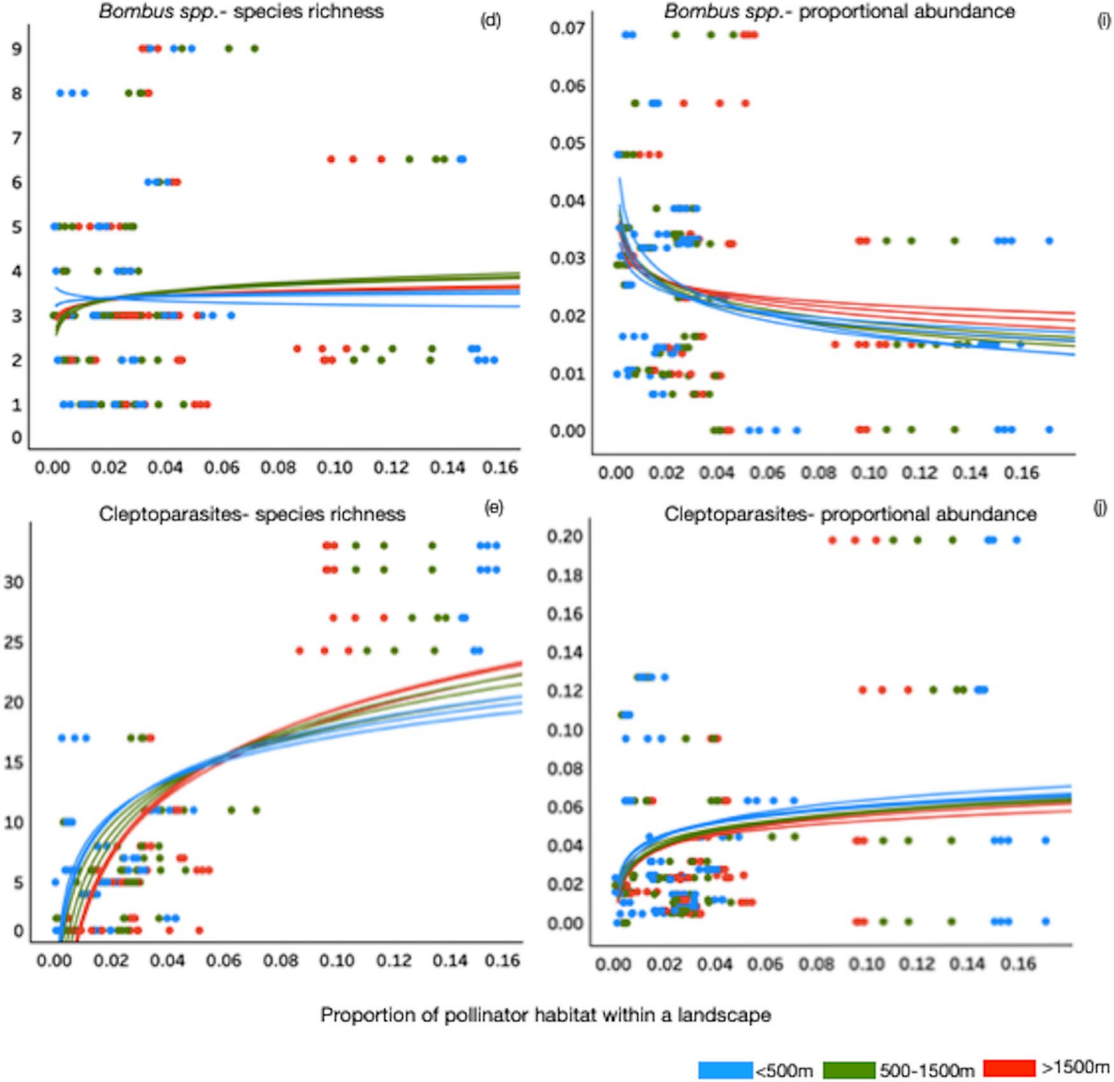
Relationship between the proportion of pollinator habitat within a landscape in Southern Ontario and community parameters, species richness and proportional abundance of solitary ground nesting bee species (**a**, **f**); social ground nesters (**b**, **g**); cavity nesters (**c**, **h**); bumblebees (*Bombus* spp.) (**d**, **i**); cleptoparasites (**e**, **j**). Logarithmic trendlines in blue are for spatial scale < 500 m, green trendlines represent spatial scale between 500 and 1500 m, and red trendlines are distances > 1500 m. Regression coefficients and *p* values associated with each spatial scale trendline and functional guild community parameter can be found in Table 1.

We found robust support for positive logarithmic relationships between the proportion (amount) of suitable habitat within a landscape and bee species richness across all functional guilds at all tested spatial scales, except for bumblebees (*Bombus* spp.) at foraging distances < 500 m (Fig. 3d, blue line; Table 1). In contrast, we found no significant logarithmic relationships between the proportional abundance of species in functional guilds and the amount of suitable habitat with the landscape at any foraging distances (Fig. 3f–j; Table 1).

**Table 1:** Species richness and proportional abundance, for each of the five functional guilds: solitary ground nesters, social ground nesters, cavity nesters, bumblebees (*Bombus* spp.) and cleptoparasites in relation to the total amount of pollinator habitat at each spatial scale.

F/(m)	Solitary ground				Social ground				Cavity				*Bombus* spp.				Cleptoparasite			
	Richness		Abundance		Richness		Abundance		Richness		Abundance		Richness		Abundance		Richness		Abundance	
	R^2^	*p*	R^2^	*p*	R^2^	*p*	R^2^	*p*	R^2^	*p*	R^2^	*p*	R^2^	*p*	R^2^	*p*	R^2^	*p*	R^2^	*p*
250	**0.26**	**0.02**	0.13	0.12	0.14	0.11	0.005	0.76	0.05	0.34	0.11	0.15	0.01	0.67	0.06	0.51	**0.37**	**0.01**	0.08	0.11
300	**0.22**	**0.04**	0.08	0.39	0.14	0.17	< 0.001	0.98	0.09	0.20	0.13	0.13	< 0.001	0.85	0.002	0.92	**0.39**	***	**0.04**	0.24
350	0.08	0.19	0.02	0.54	0.07	0.25	0.022	0.52	**0.25**	**0.02**	< 0.001	0.89	< 0.001	0.97	0.002	0.68	0.11	0.14	0.001	0.97
400	**0.30**	**0.01**	0.02	0.45	**0.29**	**0.01**	0.05	0.30	**0.39**	***	0.002	0.87	0.10	0.16	0.01	0.53	**0.42**	***	< 0.001	0.60
450	**0.29**	**0.01**	0.03	0.39	**0.25**	**0.01**	0.062	0.22	**0.44**	***	0.003	0.81	0.03	0.40	0.01	0.51	**0.48**	***	0.017	0.30
500	**0.29**	**0.01**	0.03	0.44	**0.24**	**0.01**	0.0004	0.92	**0.36**	***	0.04	0.31	0.009	0.65	0.03	0.33	**0.44**	***	0.012	0.28
750	**0.23**	**0.01**	0.07	0.14	**0.22**	**0.02**	0.006	0.78	**0.36**	**0.04**	**0.23**	**0.01**	< 0.001	0.95	0.002	0.71	**0.38**	***	0.05	0.09
1000	**0.29**	**0.01**	0.07	0.12	**0.32**	***	0.04	0.40	**0.17**	**0.06**	**0.18**	**0.02**	0.025	0.44	0.03	0.46	**0.33**	***	0.09	0.20
1250	**0.24**	**0.01**	0.05	0.23	**0.27**	**0.01**	0.05	0.31	**0.14**	**0.07**	0.10	0.09	0.025	0.44	0.012	0.69	**0.33**	***	< 0.001	0.32
1500	**0.22**	**0.01**	0.02	0.41	**0.18**	**0.03**	0.09	0.15	0.13	0.18	**0.25**	**0.01**	0.013	0.58	0.003	0.96	**0.30**	**0.01**	0.003	0.41
1750	**0.14**	**0.05**	0.001	0.73	0.10	0.11	0.14	0.07	0.07	0.31	**0.31**	***	0.008	0.67	< 0.001	0.92	**0.19**	**0.03**	0.004	0.73
2000	**0.27**	**0.01**	0.05	0.24	**0.28**	**0.01**	0.01	0.62	0.04	0.01	0.16	0.08	0.003	0.78	0.003	0.62	**0.42**	***	0.004	0.29

F is the spatial scale (m) from each site. Significant results are shown in bold (alpha = 0.05), *p* values < 0.005 represented by *** due to space constraints in table.

### Which habitats are most important?

**Figure 4:**
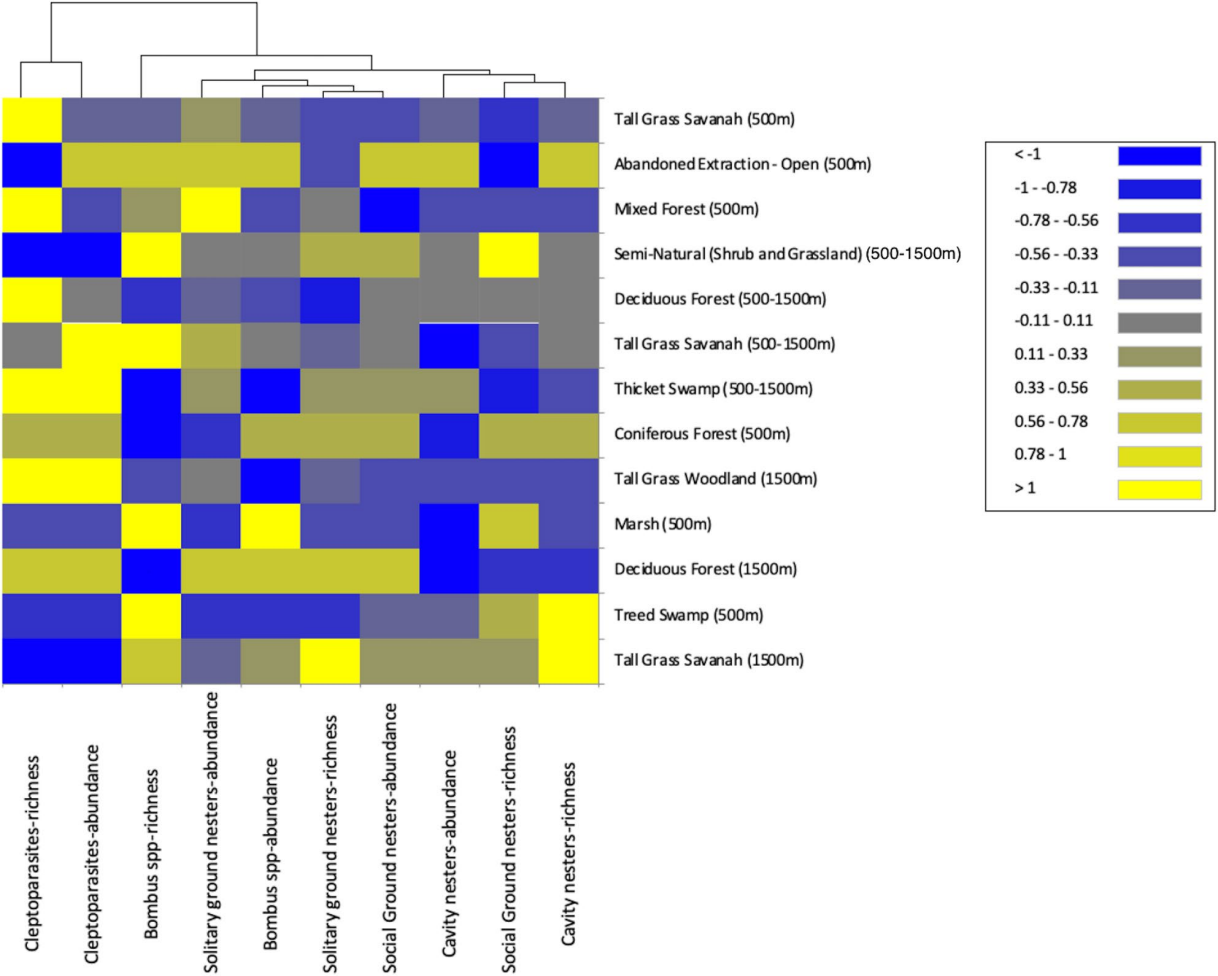
Heat map showing the most important habitat types driving key bee biodiversity metrics (species richness and proportional abundance) of five functional bee guilds: solitary ground nesters, social ground nesters, cavity nesters, bumblebees (*Bombus* spp. excluding subgenus *Psithryus*), and cleptoparasitic species (including *Bombus* subgenus *Psithryus*) at three spatial scale categories (< 500 m, 500–1500 m, and > 1500 m); lighter shades of yellow indicate most preferred habitat types at different spatial distances, darker shades of blue suggest a less desirable habitat for supporting functional guild species richness and abundance. Black cells indicate the habitat has a neutral impact on bee species richness in the landscape. Habitat similarity is characterized by similar groupings of colours, either among function guilds (horizontal rows) or across spatial distances and habitat types (vertical columns). Forested habitats represented 50 m edges of habitat. This is a filtered version of the overall heat map (Fig. S1) from which habitat types with an interquartile range of < 0.25 of significant β coefficients (habitat types) have been removed. Heat map was produced using XLSTAT v.4.1^63^.

The full heat map clearly shows a diverse range of habitat types are needed to support wild bee communities across a range of spatial scales (Fig. S1). However, to help decision-makers successfully prioritise the most important habitats to maintain, restore or create we filtered the full heat map (by removing habitat types with interquartile ranges < 0.25 for significant β coefficients) to highlight the most important habitat types in a landscape (Fig. 4). If the goal is to safeguard wider pollinator biodiversity (species richness), more habitat and distinctly different habitat types are required (Fig. 4) than if the goal is to enhance crop pollination through increasing the abundance of specific functional groups or indeed particularly important species that dominate crop flower visitation^33,34^ (Fig. 2; Table 2).

**Table 2:** The three top models testing relationships between bee functional guild (solitary ground nesters, social ground nesters, cavity nesters, bumblebees (*Bombus* spp.) and cleptoparasites) metrics (species richness and proportional abundance) and pollinator habitats (model explanatory variables). F is the spatial scale (m) from each site within the landscape; K is the number of parameters included in each candidate model; model parameters are habitat types and study year, AIC is Akaike’s Information Criterion, and *w* is Akaike weight. Bolded Rows represent functional guild abundance while non-bolded rows represent species richness.

F	K	Model variables (Ontario land classes/habitat types)	AICc	Δ AICc	*w*
**Solitary ground**					
450	6	Treed Swamp/ Deciduous Forest/ Thicket Swamp/ Consistent Foraging Crop/ Tall Grass Savanah/StudyYear	38	− 5.21	0.049
1000	5	Treed Swamp/ Forest/ Thicket Swamp/ Built Up Area Pervious/ StudyYear	45	− 10.84	0.813
2000	6	Forest/ Coniferous Forest/ Deciduous Forest/ Plantation/ Tall Grass Savanah/ StudyYear	54	− 5.32	0.051
**500**	**6**	**Coniferous Forest/ Mixed Forest/ Thicket Swamp/ Marsh/ Tall Grass Savanah/ StudyYear**	**− 109**	**− 25.29**	**0.685**
**1000**	**6**	**Treed Swamp/ Coniferous Forest/ Deciduous Forest/ Hedge Row/ Tall Grass Savanah/ StudyYear**	**− 115**	**− 3.52**	**0.000**
**1500**	**6**	**Thicket Swamp/ Built Up Area Pervious/ Abandoned Extraction Vegetated/ Tall Grass Woodland/ Tall Grass Savanah/ StudyYear**	**− 111**	**0.00**	**0.000**
**Social ground**					
350	6	Treed Swamp/ Pasture/ Forest/ Plantation/ Consistent Foraging Crop/ StudyYear	41	− 9.24	0.730
1000	6	Treed Swamp/ Deciduous Forest/ Marsh/ Built Up Area Pervious/ Consistent Foraging Crop/ StudyYear	56	− 4.00	0.053
2000	5	Treed Swamp/ Coniferous Forest/ Thicket Swamp/ Semi-Natural (Shrub and Grassland)/ StudyYear	62	− 1.42	0.015
**250**	**6**	**Treed Swamp/ Pasture/ Mixed Forest/ Plantation/ Tall Grass Savanah/ StudyYear**	**− 78**	**0.00**	**0.087**
**1000**	**6**	**Treed Swamp/ Coniferous Forest/ Plantation/ Hedge Row/ Semi-Natural (Shrub and Grassland)/ StudyYear**	**− 102**	**0.14**	**0.083**
**2000**	**3**	**Thicket Swamp/ Semi-Natural (Shrub and Grassland)/ StudyYear**	**− 104**	**− 3.25**	**0.077**
**Cavity**					
500	6	Treed Swamp/ Forest/ Semi-Natural (Shrub and Grassland)/ Consistent Foraging Crop/ Tall Grass Woodland/ StudyYear	31	− 23.56	0.934
1250	5	Treed Swamp / Thicket Swamp / Plantation / Hedge Row / StudyYear	45	− 3.75	0.000
2000	6	Forest / Coniferous Forest / Mixed Forest / Deciduous Forest / Abandoned Extraction - Open / Tall Grass Savanah	47	− 1.22	0.000
**450**	**6**	**Treed Swamp / Coniferous Forest / Marsh / Semi-Natural (Shrub and Grassland) / Consistent Foraging Crop / StudyYear**	**− 137**	**− 35.35**	**0.970**
**1250**	**6**	**Pasture / Forest / Coniferous Forest / Marsh / Abandoned Extraction Vegetated / Tall Grass Savanah**	**− 125**	**0.48**	**0.000**
**2000**	**6**	**Treed Swamp / Coniferous Forest / Deciduous Forest / Thicket Swamp / Consistent Foraging Crop / StudyYear**	**− 131**	**1.29**	**0.000**
***Bombus* species**					
250	6	Treed Swamp / Forest / Coniferous Forest / Mixed Forest / Marsh / StudyYear	− 2	0.00	0.041
1000	6	Deciduous Forest / Thicket Swamp / Semi-Natural (Shrub and Grassland) / Abandoned Extraction Vegetated / Tall Grass Savanah / StudyYear	0	− 5.54	0.657
2000	6	Pasture / Forest / Deciduous Forest / Semi-Natural (Shrub and Grassland) / Tall Grass Savanah / StudyYear	− 7	− 1.27	0.078
**400**	**6**	**Pasture / Marsh / Built Up Area Pervious / Consistent Foraging Crop / Tall Grass Woodland / StudyYear**	**− 214**	**− 34.30**	**0.829**
**750**	**6**	**Deciduous Forest / Thicket Swamp / Marsh / Built Up Area Pervious / Consistent Foraging Crop / StudyYear**	**− 219**	**0.00**	**0.000**
**2000**	**6**	**Coniferous Forest / Mixed Forest / Thicket Swamp / Abandoned Extraction - Open / Tall Grass Woodland / StudyYear**	**− 211**	**− 3.19**	**0.000**
**Cleptoparasite**					
350	5	Deciduous Forest / Semi-Natural (Shrub and Grassland) / Consistent Foraging Crop / Tall Grass Woodland / StudyYear	38	− 3.72	0.270
750	6	Deciduous Forest / Thicket Swamp / Plantation / Semi-Natural (Shrub and Grassland) / Consistent Foraging Crop / StudyYear	46	0.00	0.042
2000	6	Pasture / Semi-Natural (Shrub and Grassland) / Built Up Area Pervious / Tall Grass Woodland / Tall Grass Savanah / StudyYear	48	− 3.85	0.289
**500**	**2**	**Deciduous Forest / Tall Grass Woodland**	**− 159**	**− 32.36**	**0.971**
**1250**	**6**	**Treed Swamp / Coniferous Forest / Thicket Swamp / Hedge Row / Semi-Natural (Shrub and Grassland) / Tall Grass Savanah**	**− 168**	**− 1.70**	**0.000**
**2000**	**2**	**Tall Grass Woodland / Tall Grass Savanah**	**− 166**	**0.00**	**0.000**

F is the spatial scale (m) from each site within the landscape; K is the number of parameters included in each candidate model; model parameters are habitat types and study year, AIC is Akaike’s Information Criterion, and *w* is Akaike weight. Rows in grey represent functional guild abundance while non-coloured rows represent species richness.

Partial regression coefficients (*β*_1_) for habitat covariates (independent variables) used in models of best fit for each spatial category were reported as a way to assess the importance of habitat types for target bee community parameters for each functional guild (Tables S8–9). The importance of conserving sensitive lands, such as tallgrass woodlands and wetland habitat, for bee species appeared to far outweigh other habitat types such as hedgerows and semi-natural habitat (Fig. 4; Table 2; Tables S8–9). Wetland and forest edge habitats were significant predictors of species richness in all bee groups across a range of foraging distances (Fig. 4).

We found a number of habitat types were not highlighted among the six most important for any functional guilds within our spatial categories (Tables S8–9). Abandoned extraction-vegetated, plantations, and treed sand dunes were not found to be important drivers of functional guild species richness or abundance at the most local scales (< 500 m: Tables S8–9). Abandoned extraction-open and coniferous forest habitats were not the most important for supporting abundance of any functional guild, whereas built-up pervious habitats were not important parameters for species richness for any guild (Tables S8–9).

Our results show both abandoned extractions-open and treed sand dunes to be less important than other habitat types for maintaining species richness and abundance of functional guilds at 750–1250 m (Tables S8–9). Built-up pervious, and total pollinator habitat were not among the most important covariates for any functional guild abundance, whereas mixed forests, pastures, and plantations appeared not to be the most important predictors of functional guild species richness in our analyses (Tables S8–9).

Abandoned extraction-open, tallgrass savannah and treed swamps appeared not to be important predictors for any functional guilds at the largest spatial scales (> 1500 m: Tables S8–9). Furthermore, at spatial scales > 1500 m, four different habitats (built-up previous, deciduous forest, consistent crop, and semi-natural) were not selected in any of our best fitting candidate models for functional guild abundance, and two more habitats (marsh and mixed forest) were not selected as supporting species richness for any functional guild (Table S8).

## Discussion

Existing information on the provision of pollinator habitat in a landscape suggests its value is context dependent, and that there comes a point when adding more habitat provides little or no further measurable pollinator biodiversity benefits^28^ (Fig. 1b). We tested this predicted law of diminishing returns between the maximum species richness supported when different amounts of suitable habitat are found in the landscape, closely following the species-area relationship. Our analyses provided robust support for such positive logarithmic relationships between the proportion (amount) of suitable habitat within a landscape and bee species richness across all functional guilds, except for bumblebees (*Bombus* spp.) at spatial scale < 500 m (Fig. 3d, blue line; Table S6). We did not expect to find a significant logarithmic relationship between the proportional abundance of species in functional guilds and the amount of suitable habitat as this is more likely influenced by site level characteristics (including the availability of nesting and floral resources) at each sampling location—a view supported by previous studies in agricultural^35^ and urban habitats^12^. In fact, our results demonstrated that increasing either the availability of nesting or foraging resources at our measured spatial scales did not support increases in bee abundance for social ground nesters, cavity nesters or bumblebees *Bombus* spp*.* (Fig. 3g,h,i); but it did for solitary ground nesters and cleptoparasites (Fig. 3f,j). It is plausible that social ground nesters, cavity nesters and cleptoparasites are finding resources within a relatively shorter distance, or habitat at farther distances is less desirable (Fig. 3). Furthermore, our results also show that quite an increase of species richness and abundance can be achieved with much less habitat in the landscape (Fig. 3), however, conservation strategies aiming for landscapes achieving less than maximum species richness/abundance would be significantly detrimental to ensuring ecosystems and their services are fully protected.

Given models of best fit were different at all spatial scales (Table 2; Tables S8–9), our results suggest that conservation strategies to support wider bee biodiversity should preserve 11.6–16.7% of the land area as suitable habitats within a North American landscape (Fig. 2; 750–1250 m). Current policy recommendations suggest to conserve 4.5% of habitat to support pollinators in Ontario, Canada^16^. Compared to our results (that assume communities sampled from 34 wild bee surveys are healthy and sustainable), this policy substantially under-estimates the amount of habitat needed to support diverse bee communities and safeguard the pollination services they provide to crops and wild plants by 2.6–3.7 times. Any strategies aiming to safeguard pollinator biodiversity using targets below our evidence-based recommendations will likely provide insufficient habitat area for wild bees.

Many of the identified pollinator species-at-risk in North America are bumblebees^36,37^. Given that these major crop pollinators showed considerable preferences for habitat between 750 and 1250 m in our study (250–1000 m in the UK^25^), we suggest that implementing agri-environmental conservation schemes in North American landscapes that focus on ensuring natural/agricultural pollination resources at habitat distances of < 750 m will likely miss opportunities to enhance pollination services provided by wild *Bombus* species (Fig. 2a,b). The importance of conserving sensitive lands, such as tallgrass woodlands and wetland habitats, for bumblebee species appeared to far outweigh other habitat types such as hedge rows and semi-natural habitat (Fig. 4).

Promoting and maintaining a variety of forest edge habitats in agricultural areas where *Bombus* species and cavity nesters are the predominant crop pollinators could represent a more effective strategy to increase crop pollination services than implementing flowering field margins that may provide less varied nesting opportunities for these target groups (Fig. 4). Given that many habitat losses in North America are often the result of the conversion of natural land to agricultural uses^38,39^, and that agricultural expansion has resulted in significant loss of phylogenetic diversity in bee communities^40^, it is important that environmental policy in agricultural landscapes consider addition, restoration or creation of wetland habitats. Evidence-based conservation policies for supporting pollinators may also deliver other biodiversity benefits, for example providing suitable habitat for other beneficial arthropods (e.g., spiders and parasitoid wasps that can provide crop pest bio-control^41^), birds and other wildlife in the landscape. The ecosystem services provided by wetlands extend far beyond pollinators—wetlands increase the water table height and therefore the quantity of water available for crop irrigation, improve drinking water quality, flood mitigation and habitat for other wildlife, including other species-at-risk^42–44^.

It is critical to continue to implement wild pollinator monitoring programs and to identify specific ecological requirements for individual pollinator species before and after the implementation of conservation strategies. Such monitoring programs will be the best indicators of how populations are responding to any new or modified management practices^6,45^ at relevant spatial scales. Overall, we still know very little about the foraging patterns and flower preferences of the majority of wild bee species^46^, although some species (e.g., *Eucera* (*Peponapis*) *pruinosa* (hoary squash bee), *Nomia melanderi* (alkali bee), and common bumble bee species) are comparatively well studied^47,48^. Further studies of the foraging, nesting and other ecological requirements of other wild bee species would provide valuable bottom up information to inform conservation strategies, complimentary to the top down approach we have taken here.

In the face of evidence that intensive landscape management can severely limit the diversity and extent of habitat to support wild pollinators^3,5^, global conservation policies must not under-estimate what pollinators actually need to survive and thrive. Our results provide clear-cut habitat prescriptions to support specific conservation needs for wild bees. As a society we need to have a clear understanding of the specific aims, priorities and outcomes required for pollinator conservation with regards to crop pollination, maintaining wider biodiversity or targeting key species-at-risk. Our results clearly highlight that whether supporting species richness or abundance, the wrong habitat prescription will ultimately continue to prove ineffective for safeguarding wild pollinator biodiversity and the essential ecosystem services they provide.

## Methods

### Quantifying maximum bee community biodiversity targets in a common North American landscape

Recent estimates of bee diversity suggest that the province of Ontario is home to 421 of the 927 bee species found in Canada, making Ontario a national bee biodiversity hotspot and a critical location for strong pollinator conservation policy^49,50^. Ontario is one of the few provinces or territories in Canada in which multiple survey studies of wild bees have been conducted. We compiled a database that includes data from 34 wild bee surveys conducted in Ontario between 2002 and 2014^51–57^ (Table S1) and consists of 66,343 individual bee specimen records, representing 34 of the 39 genera, and 361 species out of a possible 421 species (85.7% of the known species) recorded for the province (Table S2). Given our main objective was to assess the importance of specific habitat types for maximizing wild bee diversity and abundance, selecting studies from a range of natural and agricultural landscapes with similar sampling techniques, and species identifications of bee specimen records that were all verified was crucial to discern the importance of habitat types at different scales and across taxa^58^. We chose to use bee species richness (number of species present) and abundance (number of individuals present) as target metrics for this study as both are widely used as fundamental measures of assessing changes in biodiversity across time and space^13^. An additional benefit to using simple biodiversity metrics, like the numbers of individual bees present and the number of species recorded (rather than more derived biodiversity parameters like species diversity and evenness) is that this ensures our results are more understandable and directly relevant for policymakers, farmers, land owners, conservationists and other important stakeholders that will use this information to implement actions to promote pollinator conservation on the ground.

**Figure 5:**
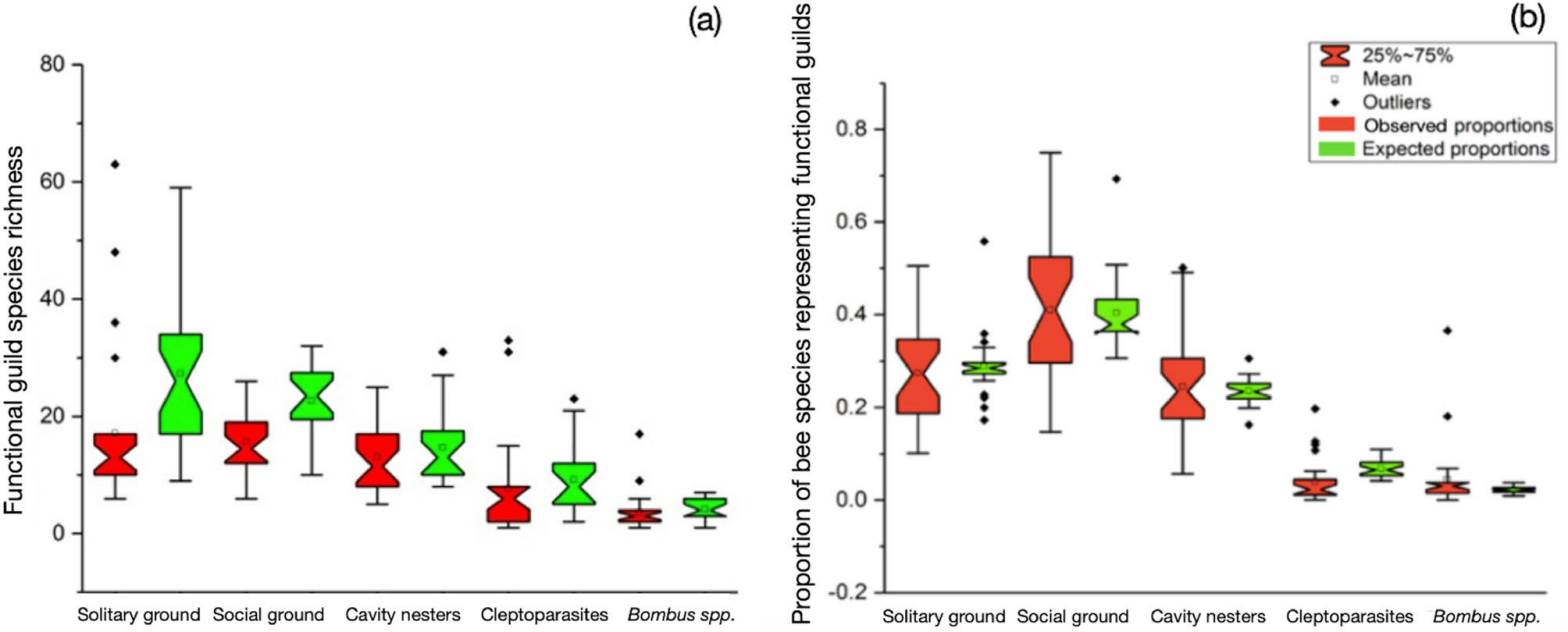
Notched-box plots depicting differences between observed and expected (maximum) (**a**) species richness and (**b**) proportion of bee species representing each functional guild: solitary ground nesters; social ground nesters; cavity nesters; cleptoparasites; and bumblebees (*Bombus* spp.). Alignment of notches denotes no significant difference between observed and expected species richness and proportional abundance for each functional guild.

In order to calculate the maximum (expected) bee biodiversity metrics (species richness and abundance), and to account for differences in sampling effort among the 34 surveys included in the database, we assessed whether our bee biodiversity metrics reported in each survey differed from random^59^. To do this, we created an expected distribution for each survey by randomly sub-sampling the same number of individuals collected in a survey from the full database of 66,271 individual specimen records. All individuals were equally weighted to allow the same chance of sampling individuals with different social structures and nesting preferences (see Supplemental materials for more information). We tested for an impact of sampling effort on bee species caught in published and unpublished studies by investigating the differences between expected and observed bee species richness and abundance for each of the 34 surveys using a mood test. We chose a mood test over other parametric tests as none of the samples used in the analyses were normally distributed (i.e. Grixti and Packer^51^, Richards et al.^52^ and Pindar^55^), yet all exhibited similar distributions. Furthermore, we chose the mood test as it is sensitive to changes in distribution. This was desirable as we expected different species to be found in different habitats. Despite high variation of observed bee species richness and abundance among survey sites and over time, these differences between observed and expected values were only significant for solitary ground nesting-, social ground nesting-, and cleptoparasite species richness (Fig. 5; Table S3). Species richness and proportional abundance for all other functional guilds (cavity nesters, *Bombus* spp.,) showed no significant differences between observed and expected bee community metrics (Table S3). These results were robust to variations in the number of times we randomly sampled bees from the overall dataset of 66,271 observations, suggesting functional roles within a pollinator community remain largely consistent across a landscape although the particular species found within specific habitat types vary^13^.

### Quantifying pollinator habitat needs

To examine the relationships between bees and habitats within a landscape, we used ground-truthed land cover data in our study. The Pollinator Habitat Baseline (PHaB) mapping layer integrates the Southern Ontario Land Resource Information System 2.1 (SOLRIS)^60^ and the Annual Crop Inventory (ACI) produced by Agriculture and Agri-Food Canada (AAFC)^61^, land cover products that provide a ground-truthed comprehensive, standardized landscape level inventory of natural, rural and urban lands at a 15-m resolution (please refer to Hogg and Jones^17^ for more detailed information on criteria for selecting high quality pollinator habitats in Ontario). Using this data was critical as it provided greater confidence that habitat type designations from map datasets are realistic descriptions of habitats types (and critically the resources they provide to pollinators) on the ground. Pollinator habitat within a landscape at each of the 34 bee survey locations was quantified using the PHaB mapping layer for the province of Ontario. The total extent (amount) of each of the 24 different pollinator habitat classes (defined by Ontario land classes (Tables S4, S5)) was quantified in non-overlapping buffer rings with radii 250, 300, 350, 400, 450, 500, 750, 1000, 1250, 1500, 1750 and 2000 m centred around the 34 survey sites at which bee species were surveyed and collected. Habitat estimates from each of these areas were then categorized into three distinct spatial range categories: < 500 m, 500–1500 m, and > 1500 m from the survey location. Calculations of the extent of pollinator habitats were conducted for each of the 12 radii separately, and were not initially added across the twelve radius distances. That is, the extents of habitats within each foraging radius were not cumulative with increasing distance from the survey site to ensure the same patches of habitat were not included multiple times into analyses.

In our study, we defined pollinator habitat as land that meets all of the forage, nesting and hibernation requirements of wild bee species. Therefore, to determine the total amount of habitat needed to maintain species richness and abundance (target bee community metrics) for each functional guild (solitary ground nesters, social ground nesters, cavity nesters, *Bombus* spp. and cleptoparasites), we calculated the proportion of pollinator habitat found from the overall area at each spatial scale (the radius of the circle centred on the study site location) in relation to the target bee community metric calculated from resampling the 34 bee surveys (Fig. 1b). The amount of habitat needed to maintain bee community metric at each spatial scale for each functional guild was calculated separately using Eq. (1):

1 $$ln\left( {x_{i} } \right) = \frac{{E\left( {a, \, r} \right) - \beta_{0} }}{{\beta_{1} }}$$

where *E(a, r)* is the expected proportional abundance (*a*) and species richness (*r*) of functional guilds, *β*_0_ is the slope of each trendline at each spatial scale (circles with radius 250 m, 300 m, 350 m, 400 m, 450 m, 500 m, 750 m, 1000 m, 1250, 1500 m, 1750, or 2000 m centred on each study site), *β*_1_ is the partial regression coefficient, and *x*_*i*_ are covariates (the amount of habitat at each spatial scale). We examined the shape of this relationship between the cumulative number of bee species supported when different amounts of suitable habitat are found in the landscape (closely following a species-area relationship) to find the point at which further additional habitat area no longer enhanced species richness—a law of diminishing returns (Figs. 1b, 3, Table 1).

### Statistical analyses

We explored potential correlations among the measured habitat covariates at the maximum foraging radius (2 km) using Spearman’s rank correlation coefficients (r_s_). Generally where r_s_ coefficients between two variables exceed 0.7 they are considered to have a strong relationship and should be removed from the analyses^62^. In our analyses there were correlations between habitat types, but while none of the r_s_ values were higher than 0.7, many were significant (*p* < 0.05: Table S6). The relationship between total pollinator habitat and several other habitat types, especially treed swamp were strongly associated (r_s_ = 0.68, *p* < 0.05; Table S6). A stronger relationship between total pollinator habitat and individual habitat types was expected as total habitat incorporates each individual habitat type. The correlation between total pollinator habitat and several habitat types was greater in comparison to other reported relationships (Table S6), thus, to be conservative we removed this parameter (total pollinator habitat) from our analyses.

To test which habitats were most important for each bee functional guild we used generalized linear mixed models (GLMMs), to explore the relationship between the amount of habitat of each habitat type (explanatory fixed variables) and species richness and proportional abundance separately at each radius distance (i.e., 5 functional guilds × 12 radius distances × 2 community parameters = 120 GLMMs in total; Table S7) using a GLMM structure with poisson error distribution with a log link, where *E(Y)* is expected response variables (species richness and proportional abundance at each radius distance), *n* is the number of variables included in the model; *β*_*n*_ are the partial regression coefficients; *X*_*n*_ are the covariates (habitat types), and *ϵ* represents the residuals. Furthermore, the Newey-West covariance correction (lag = 1) was also applied in our models to account for heteroscedasticity and autocorrelation of habitat types.

To account for study/survey differences in sampling methods and variation across multiple years, our models also included random effects for study/year. Delta AICc (∆AICc), AICc, and AICc weights (*w*) of candidate models were used to select the best fitting model within each spatial category (< 500 m, 500–1500 m, > 1500 m) shown in bold in Table S7 as they provided a better understanding of the relative effects of each of the modelled variables^23^. Both functional guild abundance and species richness were log-transformed using ln [a + 1, r + 1] prior to analyses to account for some studies/years having values of species richness and abundance of zero.

Although 24 different pollinator habitat types were found in the landscape (Tables S4, S5), we parameterized our global models for each functional guild to test for main effects of the six most influential habitat types on bee biodiversity metrics (i.e., species richness and abundance). Six habitats were chosen as it represents the lowest number of habitats that were found within a 2 km radius of a bee survey (Table S1). Prioritising the most important (six) habitat types in this way provides end users with a more targeted set of habitat recommendations to meet their specific conservation needs. We did not include interactions between different habitat covariates in our models due to a lack of biological justification for reporting statistical interactions among habitats, that may or may not be within close proximity of one another in a landscape. Partial regression coefficients (*β*_*1*_) and 95% confidence intervals (CIs) for habitat covariates (independent variables) used in the best fitting models at each spatial category were reported (significance was reported if CIs did not include zero) as a way to assess the importance of habitat types for target bee community metrics for each functional guild (Tables S8, S9).

To explore the extent of correlation between habitat types among functional guilds and across spatial scales, we clustered partial regression coefficients of significant habitat types using ascendant hierarchical clustering based on Euclidian distances, then mapped these using a heat map function to visually report results to be more easily accessible for all end-users (e.g., academics, conservation practitioners, farmers, and policymakers) wanting to interpret the results for conservation and maintenance of habitat for wild bee species (Fig. S1). We used XLSTAT and R 3.0.2 functions to run all statistical analyses^63,64^.

## Supplementary Information

Supplementary Information.

## Data Availability

Most bee and GIS dataset generated and analysed during the current study are from already published studies and available online. Bee datasets are available from: Grixti and Packer^51^ (https://doi.org/10.4039/n05-034); Richards, et al.^52^ (https://doi.org/10.4039/n11-010); Colla, et al.^53^ (https://digitalcommons.lmu.edu/cate/vol2/iss1/4); Taylor and Catling^54^ (https://doi.org/10.22621/cfn.v125i4.1258); Pindar^55^ (https://yorkspace.library.yorku.ca/xmlui/handle/10315/31967); James^56^ (https://curve.carleton.ca/system/files/etd/c58baa1b-fc25-4d99-a79a-b10a4f8d9b66/etd_pdf/87c7903a1c5b9dd810dbb32c47257e4a/james-nativebeediversityinconventionalorganichedgerows.pdf); Andrachuk^57^ (https://uwspace.uwaterloo.ca/handle/10012/8254); and GIS datasets are from: OMNRF^60^ (https://www.javacoeapp.lrc.gov.on.ca/geonetwork/srv/en/main.home?uuid=635529ce-2639-46f8-9fc2-43fbdd68aad1), AAFC^61^ (http://www.agr.gc.ca/atlas/supportdocument_documentdesupport/annualCropInventory/en/ISO19131_AAFC_Annual_Crop_Inventory_Data_Product_Specifications.pdf). Two datasets are not publicly available due to several sites being located on private land and landowners do not want exact locations shared but bee lists are available from the corresponding author upon reasonable request.
