# Supplementary material for: Safeguarding pollinators requires specific habitat prescriptions and substantially more land area than suggested by current policy
Source: Sci Rep. 2023 Mar 21;13:1040. doi: 10.1038/s41598-022-26872-x (PMC10030592; doi:10.1038/s41598-022-26872-x)
Supplement: Supplementary file 1 — Supplementary Information. [file 41598_2022_26872_MOESM1_ESM.docx]

**Safeguarding pollinators requires specific habitat prescriptions and substantially more land area than suggested by current policy**

**Supplemental Materials**

Alana Pindar^1,2†^, Nigel E. Raine^2^

^†^Corresponding author. E-mail: Alana_Pindar@cbu.ca

^1^ School of Science and Techonology, Cape Breton University, Sydney NS, B1P 6L2, Canada

^2^ School of Environmental Sciences, University of Guelph, Guelph ON, N1G 2W1, Canada

**Creating a wild bee database for Ontario**

Our database includes no species records from five other genera known from other records in the province: *Svastra* spp. (Apidae); *Pthilothrix bombiformis* (Cresson) (Apidae); *Dieumonia* spp. (Halictidae); *Dufourea* spp. (Halictidae); and *Dianthidium* spp. (Megachilidae) (Table S2)^63^. All bees reported in each study/survey were included in the database with the exception of a small number of *Nomada* specimens. These specimens were flagged and removed from the database due to their unreliable or unverified identification information (for example, “*Nomada* spp*./* spp1/ Form a/ Form d/ Form e”) to avoid confusion or duplication of individual or species records.

While functional diversity is technically defined as the diversity of species’ traits, this term is also often used to represent the diversity of species’ ecological functions and/or guilds within an ecosystem^64,65^. Unlike traditional metrics used to measure species richness and diversity, metrics of functional diversity provide a mechanistic link between species and environmental factors^65^ as species are assigned to a guild based on *a priori* expectations of taxonomic, morphological, or physiological traits^66^. Therefore, using functional guilds to assess the status of bees in diverse habitats in Ontario provided us greater insight into the ecological habitat requirements of different bee species compared to examining bee communities as a whole^67,68^. Re-sampled bee lists from each of the 34 studies were divided into the following guilds for analysis: (1) solitary ground nesters, (2) social ground nesters, (3) cavity nesters, (4) bumblebees or *Bombus* spp. (except subgenus *Psithyrus*), and (5) clepto- and social parasites (including *Bombus* subgenus *Psithyrus*). Ground nesters were split into two guilds because social bee colonies can contain many individuals per nest and are active over a much longer period of the year, whereas most solitary bees have only one female per nest and are generally active for only a few weeks^68,69^. **Solitary ground nesters** (including species in the genera *Agapostemon*, *Andrena,* *Colletes, Lasioglossum* subgenus *Dialictus* [other than the species listed under cavity nesters below; although many *Dialictus* are eusocial, those we found in this study are solitary^70^], and *Lasioglossum* subgenus *Lasioglossum*) and **Social ground nesters** (including species in the genera *Augochlorella*, *Halictus*, and *Lasioglossum* subgenus *Evylaeus*) generally prefer open habitats, often those with dry sandy soils. **Cavity nesters** (including species in the genera *Augochlora,* *Ceratina*, *Hoplitis*, *Hylaeus, Lasioglossum (D.) cressonii* Robertson*, Lasioglossum (D.) semicaeruleum* Cockerell, and *Osmia*) nest in pithy plant stems, rock cavities and abandoned beetle burrows in wood, usually using pre-existing cavities^71^. ***Bombus* spp.** (bumblebees) were placed in a guild on their own, as they are social cavity nesters. Because species within *Bombus* subgenus *Psithyrus* are social parasites, the *Bombus* guild refers only to the non-parasitic species. **Cleptoparasites** (species in the genera *Nomada* and *Sphecodes*) as well as social parasites (*Bombus* subgenus *Psithyrus* spp.) were united under the guild “cleptoparasites” as they are all bees that lay their eggs in nests of other bees.


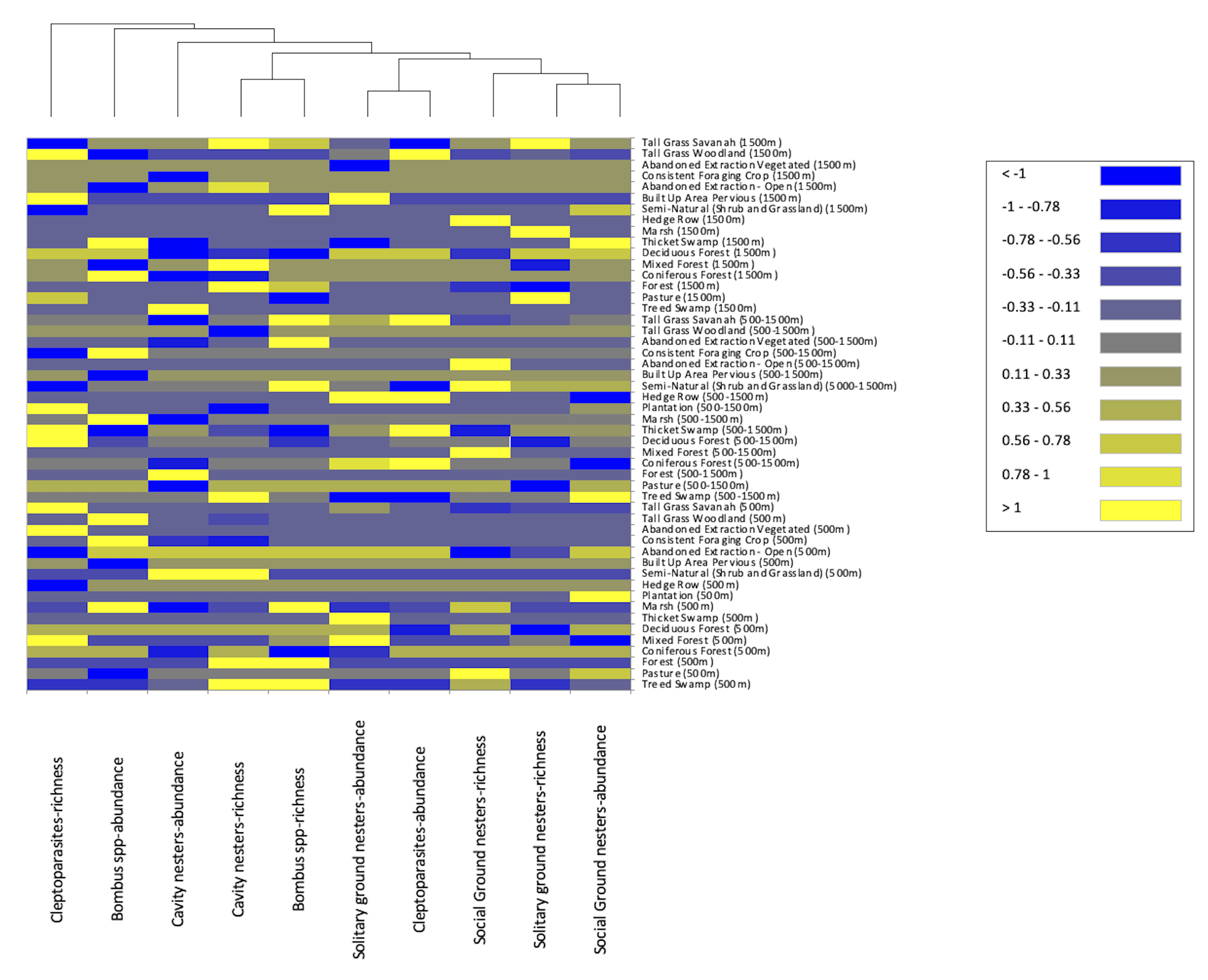


**Figure S1.** Heat map of habitat model parameters and their importance for maintaining expected bee biodiversity metrics: species richness and proportional abundance of functional guilds: solitary ground nesters, social ground nesters, cavity nesters, bumblebees (*Bombus* spp.), and cleptoparasitic species at foraging distance categories (<500m, between 500-1500m, and >1500m). Lighter shades of yellow indicate a greater importance for the habitat at different spatial distances, where darker shades of blue suggest a less desirable habitat for supporting functional guild species richness and abundance. Habitat similarity is characterized by groupings of a colour: among function guilds (horizontal rows), and across spatial distances and habitat types (vertical columns). Forested habitats represented 50m of habitat edges. Grey cells imply the habitat has a neutral impact on bee species richness and abundance in the landscape. Heat map was produced using XLSTAT v.4.1^81^.

**Table S1.** Bee survey year, number of sites sampled, sampling method, number of sites and the total number of habitats represented as the percentage of the total habitats in Ontario, and the number of total sites represented in this study from each published/unpublished study used in analyses. Note- although 8 sites were surveyed in Richards et al. (2003), habitats types did not differ among surveys/scale in your analyses and are considered one.

| **Study** | **Survey year** | **Sampling**  **method** | **Number of individuals/studies** | **Sites (names as in publications if no names then sites numbered)** | **Total number of habitat types found within surveys** | **Habitat types in surveys as percentage of ON habitats (%)** | **Habitat in surveys percentage (%)** |
| --- | --- | --- | --- | --- | --- | --- | --- |
| Grixti and Packer | 2002-2003 | Sweep/pan trapping | 10399 | Gschwendtner | 12 | 48.00 | 66.67 |
|  | 2002  ^(unpublished data)^ |  | 10078 | 1 | 11 | 44.00 | 61.11 |
| Richards et al. (2003) | 2003 | Pan trapping/sweep/aerial | 13315 | 1 | 11 | 44.00 | 61.11 |
|  |  |  |  | 2 | 11 | 44.00 | 61.11 |
|  |  |  |  | 3 | 11 | 44.00 | 61.11 |
|  |  |  |  | 4 | 11 | 44.00 | 61.11 |
|  |  |  |  | 5 | 11 | 44.00 | 61.11 |
|  |  |  |  | 6 | 11 | 44.00 | 61.11 |
|  |  |  |  | 7 | 11 | 44.00 | 61.11 |
|  |  |  |  | 8 | 11 | 44.00 | 61.11 |
| Colla et al. (2009) | 2004-2006 | Pan trapping | 342 | GR1 | 11 | 44.00 | 61.11 |
|  |  |  | 101 | GR2 | 11 | 44.00 | 61.11 |
|  |  |  | 373 | F1 | 13 | 52.00 | 72.22 |
|  |  |  | 309 | F2 | 13 | 52.00 | 72.22 |
|  |  |  | 118 | W | 7 | 28.00 | 38.89 |
|  |  |  | 299 | L | 9 | 36.00 | 50.00 |
| Taylor & Catling (2011) | 2007 | Pan trapping | 201 | Burned | 11 | 44.00 | 61.11 |
|  |  |  | 5 | Unburned | 11 | 44.00 | 61.11 |
| Pindar (2014) | 2007-2010 | Pan trapping | 3712 | Turkey | 11 | 44.00 | 61.11 |
|  |  |  | 8955 | Pinery | 12 | 48.00 | 66.67 |
|  |  |  | 13034 | LEF | 12 | 48.00 | 66.67 |
| James (2011) | 2009 | Pan trapping | 66 | James C1 | 12 | 48.00 | 66.67 |
|  |  |  | 104 | James C2 | 6 | 24.00 | 33.33 |
|  |  |  | 158 | James C3 | 6 | 24.00 | 33.33 |
|  |  |  | 224 | James C4 | 6 | 24.00 | 33.33 |
|  |  |  | 216 | James C5 | 9 | 36.00 | 50.00 |
|  |  |  | 191 | James C7 | 9 | 36.00 | 50.00 |
|  |  |  | 189 | James C8 | 12 | 48.00 | 66.67 |
|  |  |  | 105 | James C9 | 11 | 44.00 | 61.11 |
|  |  |  | 66 | James C10 | 12 | 48.00 | 66.67 |
|  |  |  | 67 | James O1 | 11 | 44.00 | 61.11 |
|  |  |  | 170 | James O2 | 8 | 32.00 | 44.44 |
|  |  |  | 173 | James O3 | 7 | 28.00 | 38.89 |
|  |  |  | 474 | James O4 | 8 | 32.00 | 44.44 |
|  |  |  | 337 | James O5 | 8 | 32.00 | 44.44 |
|  |  |  | 240 | James O7 | 9 | 36.00 | 50.00 |
|  |  |  | 125 | James O8 | 11 | 44.00 | 61.11 |
|  |  |  | 205 | James O9 | 10 | 40.00 | 55.56 |
|  |  |  | 123 | James O10 | 12 | 48.00 | 66.67 |
| Andrachuk (2014) | 2014 | Pan trapping | 1060 | RARE | 8 | 32.00 | 44.44 |
|  |  |  | 809 | RBG | 13 | 52.00 | 72.22 |
| **Total individuals, sites; mean no. of habitats and % of habitats** | | | **66343** | **34** | **10.24** | **40.98** | **56.91** |

**Table S2.** Distribution of the 361 wild bee species (from 66,343 individual records) among 39 genera and comparisons of database generic richness with the Ontario bee species list generated from published and unpublished studies ^72-78^.

| Family | Genus | Number of species | | Percentage of Ontario species in database |
| --- | --- | --- | --- | --- |
|  |  | Database | Ontario |  |
| Andrenidae | *Andrena* spp. | 64 | 75 | 85% |
|  | *Calliopsis* spp. | 1 | 1 | 100% |
|  | *Perdita* spp. | 4 | 6 | 67% |
|  | *Pseudopanurgus* spp. | 2 | 2 | 100% |
| Apidae | *Anthophora* spp. | 2 | 2 | 100% |
|  | *Bombus* spp. | 19 | 25 | 76% |
|  | *Ceratina* spp. | 4 | 4 | 100% |
|  | *Epeolus* spp. | 8 | 12 | 67% |
|  | *Eucera spp.* | 1 | 1 | 100% |
|  | *Holcopasites* spp. | 1 | 1 | 100% |
|  | *Melissodes* spp. | 11 | 11 | 100% |
|  | *Nomada* spp. | 34 | 34 | 100% |
|  | *Ptilothrix* spp. | 0 | 1 | - |
|  | *Svastra* spp. | 0 | 1 | - |
|  | *Triepeolus* spp. | 2 | 9 | 22% |
|  | *Xylocopa* spp. | 1 | 1 | 100% |
| Colletidae | *Colletes* spp. | 4 | 17 | 24% |
|  | *Hylaeus* spp. | 12 | 14 | 86% |
| Halictidae | *Agapostemon* spp. | 4 | 4 | 100% |
|  | *Augochlora* spp. | 1 | 1 | 100% |
|  | *Augochlorella* spp. | 1 | 1 | 100% |
|  | *Augochloropsis* spp. | 1 | 1 | 100% |
|  | *Dieunomia* spp. | 0 | 1 | - |
|  | *Duforea* spp. | 0 | 5 | - |
|  | *Halictus* spp. | 4 | 4 | 100% |
|  | *Lasioglossum* spp. | 66 | 74 | 89% |
|  | *Sphecodes* spp. | 22 | 23 | 96% |
| Megachilidae | *Anthidellum* spp. | 1 | 1 | 100% |
|  | *Anthidium* spp. | 1 | 3 | 33% |
|  | *Chelostoma* spp. | 1 | 3 | 33% |
|  | *Coelioxys* spp. | 3 | 10 | 30% |
|  | *Chelostoma* spp. | 1 | 3 | 33% |
|  | *Dianthidium* spp. | 0 | 1 | - |
|  | *Heriades* spp. | 3 | 3 | 100% |
|  | *Hoplitis* spp. | 5 | 7 | 71% |
|  | *Megachile* spp. | 18 | 20 | 90% |
|  | *Osmia* spp. | 22 | 24 | 92% |
|  | *Stelis* spp. | 4 | 8 | 50% |
| Melittidae | *Macropis* spp. | 1 | 1 | 100% |
|  | **Total** | **361** | **421** | **86%** |

**Table S3.** Mood test of observed and null (expected) bee community parameters: a) species richness and b) proportional abundance. Data presented are the expected (Exp.), and observed (Obs.) values for each parameter, the percentage difference between observed and expected values, and the U statistic (critical value = 3.84) and p values for these comparisons. Significant comparisons are shown in bold.

| Functional guild | 1. Species richness | | | | | 1. Proportional abundance | | | | |
| --- | --- | --- | --- | --- | --- | --- | --- | --- | --- | --- |
|  | Exp. | Obs. | % diff | U | P | Exp. | Obs. | % diff | U | P |
| Solitary ground | **17.15** | **27.27** | **59.01** | **17.43** | **<0.0001** | 0.29 | 0.27 | -6.90 | 0.016 | 0.8984 |
| Social ground | **15.13** | **22.70** | **50.03** | **15.01** | **<0.0001** | 0.42 | 0.41 | -2.38 | 0.019 | 0.8910 |
| Cavity nesters | 13.11 | 14.70 | 12.13 | 1.143 | 0.2850 | 0.22 | 0.25 | -13.64 | 0.018 | 0.8808 |
| *Bombus* spp. | 3.65 | 4.16 | 13.97 | 1.523 | 0.2127 | 0.04 | 0.04 | 0.00 | 0.026 | 0.8991 |
| Cleptoparasites | **7.68** | **9.17** | **19.40** | **13.24** | **0.0002** | 0.03 | 0.04 | +33.33 | 0.027 | 0.8913 |

**Table S4.** Ontario land classes found within 2 km of each of 34 bee sampling locations in Southern Ontario. Total amounts of each habitat found in sampling location and total amount in Southern Ontario from Hogg and Jones^79^.

| **Ontario Land Classes** | **Pollinator provisions from habitat** | **Total amount in Southern Ontario (ha)** | **Habitat percentage of out of total** | **Total habitat in surveys (at 2km) (ha)** | **Habitat in surveys percentage (%)** |
| --- | --- | --- | --- | --- | --- |
| Abandoned Extraction Open | Nesting | 22940 | 0.83 | 1.88 | 1.07 |
| Abandoned Extraction Vegetated | Forage & nesting | 4374 | 0.16 | 0.78 | 0.45 |
| Built Up Area Pervious (e.g. veg. parks) | Forage & nesting | 91180 | 3.28 | 9.66 | 5.52 |
| Coniferous Forest | Nesting | 234475 | 8.44 | 8.69 | 4.96 |
| Consistent Forage crop (AAFC land data) | Forage | 52509 | 1.89 | 2.25 | 1.29 |
| Deciduous Forest | Forage & nesting | 581021 | 20.90 | 39.01 | 22.29 |
| Forest | Nesting | 45672 | 1.64 | 2.41 | 1.38 |
| Hedge Row | Forage & nesting | 56741 | 2.04 | 7.28 | 4.16 |
| Marsh | Forage & nesting | 141153 | 5.08 | 4.83 | 2.76 |
| Mixed Forest | Forage & nesting | 269536 | 9.70 | 21.2 | 12.12 |
| Open Alvar | Forage & nesting | 2039 | 0.07 | - | - |
| Open Cliff and Talus | Forage | 20 | 0.00 | - | - |
| Open Sand Dune | Nesting | 698 | 0.03 | - | - |
| Open Tallgrass Prairie | Forage & nesting | 295 | 0.01 | - | - |
| Pasture | Forage & Nesting | 150000 | 5.40 | 0.23 | 0.01 |
| Plantation | Forage & nesting | 87154 | 3.14 | 6.83 | 3.9 |
| Semi-Natural | Forage & nesting | 72238 | 2.60 | 5.29 | 3.02 |
| Shrub Alvar | Forage & nesting | 699 | 0.03 | - | - |
| Tallgrass Savannah | Forage & nesting | 694 | 0.02 | 0.04 | 0.02 |
| Tallgrass Woodland | Forage & nesting | 1208 | 0.04 | 12.23 | 6.99 |
| Thicket Swamp | Forage & nesting | 118904 | 4.28 | 2.68 | 1.53 |
| Treed Alvar | Forage & nesting | 545 | 0.02 | - | - |
| Treed Cliff and Talus | Forage | 125 | 0.00 | - | - |
| Treed Sand Dune | Forage & nesting | 503 | 0.02 | 0.75 | 0.43 |
| Treed Swamp | Forage & nesting | 844695 | 30.39 | 49.182 | 28.11 |
| **Totals** |  | 2,779,418 (ha) | 100% | 175.24 (ha) | 100% |

**Table S5.** Description of the Ontario habitat types found within 2 km of each bee sampling location as determined by Lee ^80^ and Hogg and Jones ^79^.

| **Ontario Land Classes/ Habitat Type** | **Plant Community** |
| --- | --- |
| Abandoned Extraction Open | Inactive pit/quarry |
| Abandoned Extraction Vegetated | Inactive pit/quarry with tree cover ≤ 25%; shrub cover ≤ 25% |
| Built Up Area Pervious (e.g. veg. parks) | Vegetated Park area |
| Coniferous Forest | Largely continuous forest canopy composed primarily of coniferous species; includes swamps. |
| Consistent Forage crop (AAFC land data) | Open Agriculture- annual row crops Tobacco, ginseng, oilseeds, borage, camelina, canola/rapeseed, flaxseed, mustard, safflower, sunflower, soybeans, pulses, peas, beans, lentils, vegetables, tomatoes, potatoes, sugar beets, other vegetables, fruits, berries, cranberry, orchards, other fruits, vineyards, hops, nursery, buckwheat, vetch, fallow |
| Deciduous Forest | Deciduous tree species > 75% of canopy cover |
| Forest | Tree cover > 60% |
| Hedge Row | Shrub agriculture land |
| Marsh | A wetland with a mineral or peat substrate inundated by nutrient-rich water and characterized by emergent graminoid vegetation |
| Mixed Forest | forest canopy composed of both deciduous and coniferous species, - conifer tree species > 25% and deciduous tree species > 25% of canopy cover |
| Open Tallgrass Prairie | Natural areas typically have unique floras (e.g. Tallgrass Savannah), areas with a cultural legacy, typically dominated by more invasive herbaceous, shrub, and tree species; tree cover typically scattered or clumped |
| Pasture | Field dominated with herbaceous vegetation and grasses with an understory of similar material in a state of decay |
| Plantation | Treed agriculture land |
| Semi-Natural | Area containing natural plant communities but with obvious human alteration in form of maintenance |
| Tallgrass Savannah | 25% < tree cover < 35%; semi-open treed communities; natural areas typically have unique floras (e.g. Tallgrass Savannah), areas with a cultural legacy, typically dominated by more invasive herbaceous, shrub, and tree species; tree cover typically scattered or clumped |
| Tallgrass Woodland | 35% < tree cover < 60%; semi-closed treed communities; natural areas typically have unique floras (e.g. Tallgrass Woodland), areas with a cultural legacy, typically dominated by more invasive herbaceous, shrub, and tree species; tree cover more closed and shaded |
| Thicket Swamp | Tree or shrub cover > 25% - dominated by hydrophytic shrub and tree species |
| Treed Sand Dune | Tree cover > 25% tree cover varies from scattered and clumped to more continuous cover |
| Treed Swamp | A mineral-rich wetland characterized by a dense cover of deciduous or coniferous trees or shrubs - tree cover > 25%; trees > 5 m in height - deciduous tree species > 75% of canopy cover - common species include Fowl Manna Grass, Spotted Touch -me -not, Bugleweed, Skunk Cabbage, Marsh Marigold, Bedstraws and Stinging Nettles - typically fern and sedge rich |

| Ontario Land Classes/ Habitat Type | Ab.Ex. Open | Ab.Ex. Veget. | Bu. Up Area | Conif. Forest | Cons F Crop | Decid. Forest | Forest | Hedge Row | Marsh | Mixed Forest | Open Beach | Pasture | Plant. | Semi-Natural | Tallgr Savann | Tallgr Wood | Thick.Swamp | Tr. San Dune | Tr. Swamp. | Tot Pol Habitat |
| --- | --- | --- | --- | --- | --- | --- | --- | --- | --- | --- | --- | --- | --- | --- | --- | --- | --- | --- | --- | --- |
| Abandoned Extraction Open | **1.00** |  |  |  |  |  |  |  |  |  |  |  |  |  |  |  |  |  |  |  |
| Abandoned Extraction Vegetated | 0.01 | **1.00** |  |  |  |  |  |  |  |  |  |  |  |  |  |  |  |  |  |  |
| Built Up Area Pervious | 0.12 | 0.17 | **1.00** |  |  |  |  |  |  |  |  |  |  |  |  |  |  |  |  |  |
| Coniferous Forest | 0.01 | 0.01 | **0.21** | **1.00** |  |  |  |  |  |  |  |  |  |  |  |  |  |  |  |  |
| Consistent Foraging Crop | 0.01 | 0.01 | 0.10 | 0.01 | **1.00** |  |  |  |  |  |  |  |  |  |  |  |  |  |  |  |
| Deciduous Forest | 0.03 | 0.05 | **0.32** | **0.37** | 0.05 | **1.00** |  |  |  |  |  |  |  |  |  |  |  |  |  |  |
| Forest | 0.01 | 0.01 | **0.20** | **0.31** | 0.04 | **0.40** | **1.00** |  |  |  |  |  |  |  |  |  |  |  |  |  |
| Hedge Row | **0.16** | 0.00 | **0.36** | **0.26** | 0.04 | **0.31** | 0.14 | **1.00** |  |  |  |  |  |  |  |  |  |  |  |  |
| Marsh | 0.02 | 0.05 | **0.29** | **0.38** | 0.02 | **0.46** | **0.18** | **0.24** | **1.00** |  |  |  |  |  |  |  |  |  |  |  |
| Mixed Forest | 0.01 | 0.01 | 0.05 | **0.20** | 0.06 | 0.06 | **0.35** | 0.00 | **0.17** | **1.00** |  |  |  |  |  |  |  |  |  |  |
| Open Beach Bar | 0.05 | 0.01 | 0.04 | 0.03 | 0.00 | 0.10 | 0.02 | 0.10 | 0.00 | 0.00 | **1.00** |  |  |  |  |  |  |  |  |  |
| Pasture | 0.07 | 0.04 | 0.06 | 0.04 | **0.22** | 0.00 | 0.01 | 0.04 | 0.01 | 0.01 | **0.19** | **1.00** |  |  |  |  |  |  |  |  |
| Plantation | 0.03 | 0.11 | **0.24** | **0.22** | 0.03 | **0.31** | 0.06 | 0.06 | **0.16** | 0.15 | 0.11 | 0.00 | **1.00** |  |  |  |  |  |  |  |
| Semi-Natural | 0.02 | 0.00 | 0.01 | **0.04** | **0.18** | 0.01 | 0.01 | **0.15** | 0.01 | 0.02 | **0.16** | **0.24** | 0.01 | **1.00** |  |  |  |  |  |  |
| Tallgrass Savanah | 0.14 | 0.01 | 0.01 | 0.00 | 0.02 | 0.11 | 0.05 | 0.10 | 0.04 | 0.11 | **0.52** | 0.09 | 0.01 | 0.08 | **1.00** |  |  |  |  |  |
| Tallgrass Woodland | 0.05 | 0.01 | 0.04 | 0.03 | 0.00 | 0.10 | 0.02 | 0.10 | 0.00 | 0.00 | 0.48 | **0.19** | 0.11 | **0.16** | 0.52 | **1.00** |  |  |  |  |
| Thicket Swamp | 0.28 | 0.00 | **0.39** | **0.44** | 0.00 | **0.51** | **0.21** | **0.49** | **0.47** | 0.02 | 0.01 | 0.04 | 0.14 | 0.00 | 0.02 | 0.01 | **1.00** |  |  |  |
| Treed Sand Dune | 0.14 | 0.01 | 0.01 | 0.00 | 0.02 | 0.11 | 0.05 | 0.10 | 0.04 | 0.11 | **0.52** | 0.09 | 0.01 | 0.08 | **1.00** | 0.52 | 0.02 | **1.00** |  |  |
| Treed Swamp | 0.00 | 0.00 | 0.01 | **0.26** | 0.03 | **0.16** | **0.26** | 0.00 | 0.12 | **0.25** | 0.00 | **0.34** | **0.17** | 0.02 | 0.01 | 0.00 | 0.13 | **0.68** | **1.00** |  |
| Total Pollinator Habitat | 0.04 | 0.00 | **0.15** | **0.49** | 0.00 | **0.53** | **0.35** | 0.10 | **0.33** | **0.18** | 0.15 | **0.15** | **0.41** | 0.00 | 0.08 | 0.15 | **0.42** | **0.08** | **0.01** | **1.00** |

**Table S6.** Matrix of Spearman’s Rank correlation coefficients of relationship between habitat types. Coefficients of determination greater than 0.7 are considered strong correlations at a foraging distance of 2 km. Values in bold are different from 0 with a significance level p<0.05. Values shown as 0.00 indicate coefficients <0.005. Grey shaded cells are self-correlated habitat types (coefficient = 1.00).

**Table S7.** Candidate models testing relationships between bee functional guild (solitary ground, social ground, cavity, *Bombus* spp. and cleptoparasites) measures (species richness and proportional abundance) and pollinator habitats (model variables) in landscape and model selection statistics. F is the foraging distance (m) from each site within the landscape; K is the number of parameters included in each candidate model; model variables are habitat types, AIC is Akaike’s Information Criterion, and *w* is Akaike weight. Candidate models in bold are considered to be models of best fit in each spatial category (<500m; 750-1250m; >1500m).

| **Solitary Ground Species Richness** | | | | | | | | | | | | | | | | |  | | | | | | | | | | |  | | | |  |  |  |  |  |  |
| --- | --- | --- | --- | --- | --- | --- | --- | --- | --- | --- | --- | --- | --- | --- | --- | --- | --- | --- | --- | --- | --- | --- | --- | --- | --- | --- | --- | --- | --- | --- | --- | --- | --- | --- | --- | --- | --- |
| **F** | **K** | **Model Variables (Ontario Land Classes/ Habitat Types)** | | | | | | | | **AICc** | | | | | | | | | **Δ**  **AICc** | | | | | | | | | | | ***w*** | | | | | | |  |
| 250 | 6 | Deciduous Forest / Marsh / Hedge Row / Consistent Foraging Crop / Tall Grass Savanah / StudyYear | | | | | 43 | | | | 0.00 | | | | | | | | | | | 0.004 | | | | | | | | | |  |  |  |  |  |  |
| 300 | 6 | Deciduous Forest / Marsh / Hedge Row / Consistent Foraging Crop / Tall Grass Savanah / StudyYear | | | | | 45 | | | | 1.76 | | | | | | | | | | | 0.000 | | | | | | | | | |  |  |  |  |  |  |
| 350 | 6 | Treed Swamp / Pasture / Thicket Swamp / Consistent Foraging Crop / Tall Grass Savanah / StudyYear | | | | | 46 | | | | 2.98 | | | | | | | | | | | 0.001 | | | | | | | | | |  |  |  |  |  |  |
| 400 | 6 | Treed Swamp / Pasture / Forest / Thicket Swamp / Tall Grass Woodland / StudyYear | | | | | 39 | | | | -4.71 | | | | | | | | | | | 0.038 | | | | | | | | | |  |  |  |  |  |  |
| **450** | **6** | **Treed Swamp / Deciduous Forest / Thicket Swamp / Consistent Foraging Crop / Tall Grass Savanah / StudyYear** | | | | | **38** | | | | **-5.21** | | | | | | | | | | | **0.049** | | | | | | | | | |  |  |  |  |  |  |
| 500 | 5 | Treed Swamp / Thicket Swamp / Marsh / Consistent Foraging Crop / StudyYear | | | | | 51 | | | | 7.96 | | | | | | | | | | | 0.000 | | | | | | | | | |  |  |  |  |  |  |
| 750 | 6 | Treed Swamp / Deciduous Forest / Marsh / Semi-Natural (Shrub and Grassland) / Built Up Area Pervious / StudyYear | | | | | 56 | | | | 0.00 | | | | | | | | | | | 0.004 | | | | | | | | | |  |  |  |  |  |  |
| **1000** | **5** | **Treed Swamp / Forest / Thicket Swamp / Built Up Area Pervious / StudyYear** | | | | | **45** | | | | **-10.84** | | | | | | | | | | | **0.813** | | | | | | | | | |  |  |  |  |  |  |
| 1250 | 3 | Treed Swamp / Thicket Swamp / StudyYear | | | | | 58 | | | | 1.34 | | | | | | | | | | | 0.002 | | | | | | | | | |  |  |  |  |  |  |
| 1500 | 6 | Mixed Forest / Plantation / Abandoned Extraction - Open / Tall Grass Woodland / Tall Grass Savanah / StudyYear | | | | | 60 | | | | 0.00 | | | | | | | | | | | 0.004 | | | | | | | | | |  |  |  |  |  |  |
| 1750 | 6 | Forest / Coniferous Forest / Deciduous Forest / Plantation / Tall Grass Savanah / StudyYear | | | | | 55 | | | | -4.57 | | | | | | | | | | | 0.035 | | | | | | | | | |  |  |  |  |  |  |
| **2000** | **6** | **Forest / Coniferous Forest / Deciduous Forest / Plantation / Tall Grass Savanah / StudyYear** | | | | | **54** | | | | **-5.32** | | | | | | | | | | | **0.051** | | | | | | | | | |  |  |  |  |  |  |
| **Social Ground Species Richness** | | | | | | | | | | | | | | | | |  | | | | | | | | | | |  | | | |  |  |  |  |  |  |
| 250 | 5 | Marsh / Plantation / Consistent Foraging Crop / Tall Grass Savanah / StudyYear | | | 50 | | | 0.00 | | | | | | | | | | | | 0.007 | | | | | | | | | | | | | | |  |  |  |
| 300 | 6 | Pasture / Marsh / Plantation / Consistent Foraging Crop / Tall Grass Savanah / StudyYear | | | 49 | | | -0.38 | | | | | | | | | | | | 0.009 | | | | | | | | | | | | | | |  |  |  |
| **350** | **6** | **Treed Swamp / Pasture / Forest / Plantation / Consistent Foraging Crop / StudyYear** | | | **41** | | | **-9.24** | | | | | | | | | | | | **0.730** | | | | | | | | | | | | | | |  |  |  |
| 400 | 6 | Treed Swamp / Pasture / Coniferous Forest / Plantation / Consistent Foraging Crop / StudyYear | | | 47 | | | -2.72 | | | | | | | | | | | | 0.028 | | | | | | | | | | | | | | |  |  |  |
| 450 | 6 | Treed Swamp / Pasture / Plantation / Consistent Foraging Crop / Tall Grass Woodland / StudyYear | | | 45 | | | -4.95 | | | | | | | | | | | | 0.085 | | | | | | | | | | | | | | |  |  |  |
| 500 | 4 | Treed Swamp / Forest / Tall Grass Savanah / StudyYear | | | 58 | | | 7.78 | | | | | | | | | | | | 0.000 | | | | | | | | | | | | | | |  |  |  |
| 750 | 6 | Treed Swamp / Forest / Mixed Forest / Marsh / Plantation / StudyYear | | | 60 | | | 0.00 | | | | | | | | | | | | 0.007 | | | | | | | | | | | | | | |  |  |  |
| **1000** | **6** | **Treed Swamp / Deciduous Forest / Marsh / Built Up Area Pervious / Consistent Foraging Crop / StudyYear** | | | **56** | | | **-4.00** | | | | | | | | | | | | **0.053** | | | | | | | | | | | | | | |  |  |  |
| 1250 | 6 | Treed Swamp / Forest / Thicket Swamp / Consistent Foraging Crop / Abandoned Extraction Vegetated / StudyYear | | | 56 | | | -3.91 | | | | | | | | | | | | 0.051 | | | | | | | | | | | | | | |  |  |  |
| 1500 | 4 | Treed Swamp / Marsh / Abandoned Extraction Vegetated / StudyYear | | | 63 | | | 0.00 | | | | | | | | | | | | 0.007 | | | | | | | | | | | | | | |  |  |  |
| 1750 | 6 | Treed Swamp / Pasture / Coniferous Forest / Thicket Swamp / Abandoned Extraction Vegetated / StudyYear | | | 63 | | | -0.26 | | | | | | | | | | | | 0.008 | | | | | | | | | | | | | | |  |  |  |
| **2000** | **5** | **Treed Swamp / Coniferous Forest / Thicket Swamp / Semi-Natural (Shrub and Grassland) / StudyYear** | | | **62** | | | **-1.42** | | | | | | | | | | | | **0.015** | | | | | | | | | | | | | | |  |  |  |
| **Cavity Nesters Species Richness** | | | | | | | | | | | | | | | | |  | | | | | | | | | | |  | | |  |  |  |  |  |  |  |
| 250 | 6 | Treed Swamp / Pasture / Marsh / Built Up Area Pervious / Tall Grass Woodland / StudyYear | 54 | | | | | | 0.00 | | | | | | | | | | | | | 0.000 | | | | | | | | |  |  |  |  |  |  |  |
| 300 | 6 | Mixed Forest / Marsh / Semi-Natural (Shrub and Grassland) / Built Up Area Pervious / Tall Grass Woodland / StudyYear | 53 | | | | | | -0.69 | | | | | | | | | | | | | 0.000 | | | | | | | | |  |  |  |  |  |  |  |
| 350 | 6 | Treed Swamp / Pasture / Forest / Semi-Natural (Shrub and Grassland) / Consistent Foraging Crop / StudyYear | 50 | | | | | | -4.40 | | | | | | | | | | | | | 0.000 | | | | | | | | |  |  |  |  |  |  |  |
| 400 | 5 | Treed Swamp / Pasture / Semi-Natural (Shrub and Grassland) / Consistent Foraging Crop / StudyYear | 56 | | | | | | 1.81 | | | | | | | | | | | | | 0.000 | | | | | | | | |  |  |  |  |  |  |  |
| 450 | 6 | Treed Swamp / Pasture / Coniferous Forest / Marsh / Semi-Natural (Shrub and Grassland) / StudyYear | 36 | | | | | | -18.24 | | | | | | | | | | | | | 0.065 | | | | | | | | |  |  |  |  |  |  |  |
| **500** | **6** | **Treed Swamp / Forest / Semi-Natural (Shrub and Grassland) / Consistent Foraging Crop / Tall Grass Woodland / StudyYear** | **31** | | | | | | **-23.56** | | | | | | | | | | | | | **0.934** | | | | | | | | |  |  |  |  |  |  |  |
| 750 | 6 | Treed Swamp / Forest / Mixed Forest / Marsh / Plantation / StudyYear | 49 | | | | | | 0.00 | | | | | | | | | | | | | 0.000 | | | | | | | | |  |  |  |  |  |  |  |
| 1000 | 6 | Treed Swamp / Deciduous Forest / Thicket Swamp / Plantation / Tall Grass Woodland / StudyYear | 49 | | | | | | 0.05 | | | | | | | | | | | | | 0.000 | | | | | | | | |  |  |  |  |  |  |  |
| **1250** | **5** | **Treed Swamp / Thicket Swamp / Plantation / Hedge Row / StudyYear** | **45** | | | | | | **-3.75** | | | | | | | | | | | | | **0.000** | | | | | | | | |  |  |  |  |  |  |  |
| 1500 | 4 | Treed Swamp / Thicket Swamp / Plantation / StudyYear | 49 | | | | | | 0.00 | | | | | | | | | | | | | 0.000 | | | | | | | | |  |  |  |  |  |  |  |
| 1750 | 6 | Treed Swamp / Coniferous Forest / Marsh / Plantation / Abandoned Extraction - Open / StudyYear | 48 | | | | | | -0.62 | | | | | | | | | | | | | 0.000 | | | | | | | | |  |  |  |  |  |  |  |
| **2000** | **6** | **Forest / Coniferous Forest / Mixed Forest / Deciduous Forest / Abandoned Extraction - Open / Tall Grass Savanah** | **47** | | | | | | **-1.22** | | | | | | | | | | | | | **0.000** | | | | | | | | |  |  |  |  |  |  |  |
| ***Bombus* Species Richness** | | | | | | | | | | | | | | | | |  | | | | | | | | | | |  | | |  |  |  |  |  |  |  |
| **250** | **6** | **Treed Swamp / Forest / Coniferous Forest / Mixed Forest / Marsh / StudyYear** | | | **-2** | | | | | | | | **0.00** | | | | | | | | **0.041** | | | | | | | | | |  |  |  |  |  |  |  |
| 300 | 6 | Treed Swamp / Forest / Coniferous Forest / Marsh / Plantation / StudyYear | | | -1 | | | | | | | | 0.72 | | | | | | | | 0.029 | | | | | | | | | |  |  |  |  |  |  |  |
| 350 | 6 | Treed Swamp / Mixed Forest / Marsh / Built Up Area Pervious / Tall Grass Woodland / StudyYear | | | 11 | | | | | | | | 13.08 | | | | | | | | 0.000 | | | | | | | | | |  |  |  |  |  |  |  |
| 400 | 4 | Coniferous Forest / Deciduous Forest / Marsh / StudyYear | | | 13 | | | | | | | | 14.96 | | | | | | | | 0.000 | | | | | | | | | |  |  |  |  |  |  |  |
| 450 | 6 | Treed Swamp / Marsh / Built Up Area Pervious / Tall Grass Woodland / Tall Grass Savanah / StudyYear | | | 11 | | | | | | | | 12.50 | | | | | | | | 0.000 | | | | | | | | | |  |  |  |  |  |  |  |
| **500** | 5 | Pasture / Forest / Coniferous Forest / Plantation / StudyYear | | | 7 | | | | | | | | 9.00 | | | | | | | | 0.000 | | | | | | | | | |  |  |  |  |  |  |  |
| 750 | 6 | Treed Swamp / Pasture / Forest / Coniferous Forest / Plantation / StudyYear | | | 6 | | | | | | | | 0.00 | | | | | | | | 0.041 | | | | | | | | | |  |  |  |  |  |  |  |
| **1000** | **6** | **Deciduous Forest / Thicket Swamp / Semi-Natural (Shrub and Grassland) / Abandoned Extraction Vegetated / Tall Grass Savanah / StudyYear** | | | **0** | | | | | | | | **-5.54** | | | | | | | | **0.657** | | | | | | | | | |  |  |  |  |  |  |  |
| 1250 | 5 | Pasture / Coniferous Forest / Thicket Swamp / Plantation / StudyYear | | | 5 | | | | | | | | -1.02 | | | | | | | | 0.069 | | | | | | | | | |  |  |  |  |  |  |  |
| 1500 | 6 | Pasture / Coniferous Forest / Thicket Swamp / Plantation / Semi-Natural (Shrub and Grassland) / StudyYear | | | -6 | | | | | | | | 0.00 | | | | | | | | 0.041 | | | | | | | | | |  |  |  |  |  |  |  |
| 1750 | 6 | Pasture / Coniferous Forest / Thicket Swamp / Plantation / Semi-Natural (Shrub and Grassland) / StudyYear | | | -6 | | | | | | | | -0.13 | | | | | | | | 0.044 | | | | | | | | | |  |  |  |  |  |  |  |
| **2000** | **6** | **Pasture / Forest / Deciduous Forest / Semi-Natural (Shrub and Grassland) / Tall Grass Savanah / StudyYear** | | | **-7** | | | | | | | | **-1.27** | | | | | | | | **0.078** | | | | | | | | | |  |  |  |  |  |  |  |
| **Cleptoparasite Species Richness** | | | | | | | | | | | | | | | | |  | | | | | | | | | | |  | | |  |  |  |  |  |  |  |
| 250 | 5 | Marsh / Semi-Natural (Shrub and Grassland) / Built Up Area Pervious / Consistent Foraging Crop / StudyYear | | | | 42 | | | | | | | | | 0.00 | | | | | | | | | 0.042 | | | | | | |  |  |  |  |  |  |  |
| 300 | 6 | Treed Swamp / Marsh / Semi-Natural (Shrub and Grassland) / Built Up Area Pervious / Consistent Foraging Crop / StudyYear | | | | 39 | | | | | | | | | -2.66 | | | | | | | | | 0.159 | | | | | | |  |  |  |  |  |  |  |
| **350** | **5** | **Deciduous Forest / Semi-Natural (Shrub and Grassland) / Consistent Foraging Crop / Tall Grass Woodland / StudyYear** | | | | **38** | | | | | | | | | **-3.72** | | | | | | | | | **0.270** | | | | | | |  |  |  |  |  |  |  |
| 400 | 2 | Treed Swamp / StudyYear | | | | 72 | | | | | | | | | 30.31 | | | | | | | | | 0.000 | | | | | | |  |  |  |  |  |  |  |
| 450 | 5 | Treed Swamp / Semi-Natural (Shrub and Grassland) / Consistent Foraging Crop / Tall Grass Woodland / StudyYear | | | | 45 | | | | | | | | | 3.06 | | | | | | | | | 0.009 | | | | | | |  |  |  |  |  |  |  |
| 500 | 5 | Treed Swamp / Semi-Natural (Shrub and Grassland) / Consistent Foraging Crop / Tall Grass Woodland / StudyYear | | | | 45 | | | | | | | | | 3.11 | | | | | | | | | 0.009 | | | | | | |  |  |  |  |  |  |  |
| **750** | **6** | **Deciduous Forest / Thicket Swamp / Plantation / Semi-Natural (Shrub and Grassland) / Consistent Foraging Crop / StudyYear** | | | | **46** | | | | | | | | | **0.00** | | | | | | | | | **0.042** | | | | | | |  |  |  |  |  |  |  |
| 1000 | 6 | Coniferous Forest / Marsh / Hedge Row / Semi-Natural (Shrub and Grassland) / Tall Grass Woodland / StudyYear | | | | 48 | | | | | | | | | 1.87 | | | | | | | | | 0.017 | | | | | | |  |  |  |  |  |  |  |
| 1250 | 5 | Forest / Plantation / Semi-Natural (Shrub and Grassland) / Tall Grass Woodland / StudyYear | | | | 50 | | | | | | | | | 3.35 | | | | | | | | | 0.008 | | | | | | |  |  |  |  |  |  |  |
| 1500 | 6 | Treed Swamp / Coniferous Forest / Built Up Area Pervious / Tall Grass Woodland / Tall Grass Savanah / StudyYear | | | | 52 | | | | | | | | | 0.00 | | | | | | | | | 0.042 | | | | | | |  |  |  |  |  |  |  |
| 1750 | 6 | Forest / Coniferous Forest / Deciduous Forest / Plantation / Tall Grass Woodland / StudyYear | | | | 50 | | | | | | | | | -1.96 | | | | | | | | | 0.112 | | | | | | |  |  |  |  |  |  |  |
| **2000** | **6** | **Pasture / Semi-Natural (Shrub and Grassland) / Built Up Area Pervious / Tall Grass Woodland / Tall Grass Savanah / StudyYear** | | | | **48** | | | | | | | | | **-3.85** | | | | | | | | | **0.289** | | | | | | |  |  |  |  |  |  |  |
| **Solitary Ground Nesters Proportional Abundance** | | | | | | | | |  | | | | | | | | |  | | | | | | | | | | |  | |  |  |  |  |  |  |  |
| 250 | 6 | Mixed Forest / Deciduous Forest / Hedge Row / Consistent Foraging Crop / Tall Grass Woodland / StudyYear | | | | | -83 | | | | | | | | | 0.00 | | | | | | | | | 0.000 | | | | | | | |  |  |  |  |  |
| 300 | 6 | Forest / Mixed Forest / Deciduous Forest / Hedge Row / Tall Grass Woodland / StudyYear | | | | | -82 | | | | | | | | | 1.69 | | | | | | | | | 0.000 | | | | | | | | | | |  |  |
| 350 | 4 | Thicket Swamp / Marsh / Built Up Area Pervious / StudyYear | | | | | -96 | | | | | | | | | -12.21 | | | | | | | | | 0.001 | | | | | | | | | | |  |  |
| 400 | 6 | Forest / Thicket Swamp / Marsh / Plantation / Consistent Foraging Crop / StudyYear | | | | | -99 | | | | | | | | | -15.55 | | | | | | | | | 0.005 | | | | | | | | | | |  |  |
| 450 | 6 | Coniferous Forest / Mixed Forest / Thicket Swamp / Marsh / Tall Grass Savanah / StudyYear | | | | | -107 | | | | | | | | | -23.70 | | | | | | | | | 0.309 | | | | | | | | | | |  |  |
| **500** | **6** | **Coniferous Forest / Mixed Forest / Thicket Swamp / Marsh / Tall Grass Savanah / StudyYear** | | | | | **-109** | | | | | | | | | **-25.29** | | | | | | | | | **0.685** | | | | | | | | | | |  |  |
| 750 | 6 | Forest / Coniferous Forest / Mixed Forest / Hedge Row / Semi-Natural (Shrub and Grassland) / StudyYear | | | | | -111 | | | | | | | | | 0.00 | | | | | | | | | 0.000 | | | | | | | | | | |  |  |
| **1000** | **6** | **Treed Swamp / Coniferous Forest / Deciduous Forest / Hedge Row / Tall Grass Savanah / StudyYear** | | | | | **-115** | | | | | | | | | **-3.52** | | | | | | | | | **0.000** | | | | | | | | | | |  |  |
| 1250 | 6 | Forest / Coniferous Forest / Plantation / Hedge Row / Semi-Natural (Shrub and Grassland) / StudyYear | | | | | -111 | | | | | | | | | 0.41 | | | | | | | | | 0.000 | | | | | | | |  |  |  |  |  |
| **1500** | **6** | **Thicket Swamp / Built Up Area Pervious / Abandoned Extraction Vegetated / Tall Grass Woodland / Tall Grass Savanah / StudyYear** | | | | | **-111** | | | | | | | | | **0.00** | | | | | | | | | **0.000** | | | | | | | |  |  |  |  |  |
| 1750 | 3 | Marsh / Plantation / Tall Grass Woodland | | | | | -108 | | | | | | | | | 2.57 | | | | | | | | | 0.000 | | | | | | | |  |  |  |  |  |
| 2000 | 4 | Thicket Swamp / Marsh / Tall Grass Woodland / Tall Grass Savanah | | | | | -108 | | | | | | | | | 2.82 | | | | | | | | | 0.000 | | | | | | | | | | |  |  |
| **Social Ground Nesters Proportional Abundance** | | | | | | | | |  | | | | | | | | |  | | | | | | | | | | |  |  |  |  |  |  |  |  |  |
| **250** | **6** | **Treed Swamp / Pasture / Mixed Forest / Plantation / Tall Grass Savanah / StudyYear** | | | **-78** | | | | | | | | | **0.00** | | | | | | | | | | | | **0.087** | | | | | | | | | | | |
| 300 | 5 | Treed Swamp / Pasture / Mixed Forest / Tall Grass Savanah / StudyYear | | | -85 | | | | | | | | | -7.27 | | | | | | | | | | | | 0.087 | | | | | | | | | | | |
| 350 | 6 | Forest / Mixed Forest / Marsh / Consistent Foraging Crop / Tall Grass Savanah / StudyYear | | | -90 | | | | | | | | | -12.74 | | | | | | | | | | | | 0.087 | | | | | | | | | | | |
| 400 | 6 | Forest / Coniferous Forest / Mixed Forest / Marsh / Consistent Foraging Crop / StudyYear | | | -94 | | | | | | | | | -16.24 | | | | | | | | | | | | 0.087 | | | | | | | | | | | |
| 450 | 6 | Coniferous Forest / Mixed Forest / Thicket Swamp / Marsh / Tall Grass Savanah / StudyYear | | | -97 | | | | | | | | | -19.46 | | | | | | | | | | | | 0.086 | | | | | | | | | | | |
| 500 | 6 | Coniferous Forest / Mixed Forest / Marsh / Built Up Area Pervious / Tall Grass Savanah / StudyYear | | | -99 | | | | | | | | | -21.77 | | | | | | | | | | | | 0.085 | | | | | | | | | | | |
| 750 | 6 | Pasture / Deciduous Forest / Thicket Swamp / Marsh / Built Up Area Pervious / StudyYear | | | -102 | | | | | | | | | 0.00 | | | | | | | | | | | | 0.083 | | | | | | | | | | | |
| **1000** | **6** | **Treed Swamp / Coniferous Forest / Plantation / Hedge Row / Semi-Natural (Shrub and Grassland) / StudyYear** | | | **-102** | | | | | | | | | **0.14** | | | | | | | | | | | | **0.083** | | | | | | | | | | | |
| 1250 | 6 | Pasture / Coniferous Forest / Marsh / Plantation / Semi-Natural (Shrub and Grassland) / StudyYear | | | -105 | | | | | | | | | -3.04 | | | | | | | | | | | | 0.070 | | | | | | | | | | | |
| 1500 | 6 | Pasture / Forest / Coniferous Forest / Thicket Swamp / Abandoned Extraction Vegetated / StudyYear | | | -101 | | | | | | | | | 0.00 | | | | | | | | | | | | 0.085 | | | | | | | | | | | |
| 1750 | 3 | Thicket Swamp / Semi-Natural (Shrub and Grassland) / StudyYear | | | -101 | | | | | | | | | -0.25 | | | | | | | | | | | | 0.084 | | | | | | | | | | | |
| **2000** | **3** | **Thicket Swamp / Semi-Natural (Shrub and Grassland) / StudyYear** | | | **-104** | | | | | | | | | **-3.25** | | | | | | | | | | | | **0.077** | | | | | | | | | | | |
| **Cavity Nesters Proportional Abundance** | | | | | | | | |  | | | | | | | | |  | | | | | | | | | | |  |  |  |  |  |  |  |  |  |
| 250 | 6 | Treed Swamp / Marsh / Plantation / Semi-Natural (Shrub and Grassland) / Built Up Area Pervious / Consistent Foraging Crop | | | -102 | | | | | | | | | 0.00 | | | | | | | | | | | | | 0.000 | | | | | | | | |  |  |
| 300 | 6 | Treed Swamp / Forest / Marsh / Plantation / Semi-Natural (Shrub and Grassland) / Consistent Foraging Crop | | | -104 | | | | | | | | | -1.89 | | | | | | | | | | | | | 0.000 | | | | | | | | |  |  |
| 350 | 6 | Treed Swamp / Marsh / Semi-Natural (Shrub and Grassland) / Built Up Area Pervious / Consistent Foraging Crop / StudyYear | | | -125 | | | | | | | | | -22.80 | | | | | | | | | | | | | 0.002 | | | | | | | | |  |  |
| 400 | 6 | Treed Swamp / Marsh / Semi-Natural (Shrub and Grassland) / Consistent Foraging Crop / Tall Grass Savanah / StudyYear | | | -119 | | | | | | | | | -17.29 | | | | | | | | | | | | | 0.000 | | | | | | | | |  |  |
| **450** | **6** | **Treed Swamp / Coniferous Forest / Marsh / Semi-Natural (Shrub and Grassland) / Consistent Foraging Crop / StudyYear** | | | **-137** | | | | | | | | | **-35.35** | | | | | | | | | | | | | **0.970** | | | | | | | | |  |  |
| 500 | 6 | Thicket Swamp / Marsh / Semi-Natural (Shrub and Grassland) / Consistent Foraging Crop / Tall Grass Woodland / StudyYear | | | -130 | | | | | | | | | -28.30 | | | | | | | | | | | | | 0.029 | | | | | | | | |  |  |
| 750 | 6 | Pasture / Deciduous Forest / Marsh / Consistent Foraging Crop / Tall Grass Woodland / StudyYear | | | -125 | | | | | | | | | 0.00 | | | | | | | | | | | | | 0.000 | | | | | | | | |  |  |
| 1000 | 6 | Pasture / Deciduous Forest / Marsh / Hedge Row / Tall Grass Woodland / StudyYear | | | -127 | | | | | | | | | -2.10 | | | | | | | | | | | | | 0.000 | | | | | | | | |  |  |
| **1250** | **6** | **Pasture / Forest / Coniferous Forest / Marsh / Abandoned Extraction Vegetated / Tall Grass Savanah** | | | **-125** | | | | | | | | | **0.48** | | | | | | | | | | | | | **0.000** | | | | | | | | |  |  |
| 1500 | 6 | Forest / Coniferous Forest / Mixed Forest / Marsh / Hedge Row / Tall Grass Savanah | | | -133 | | | | | | | | | 0.00 | | | | | | | | | | | | | 0.000 | | | | | | | | |  |  |
| 1750 | 6 | Pasture / Forest / Coniferous Forest / Deciduous Forest / Marsh / Plantation | | | -128 | | | | | | | | | 4.94 | | | | | | | | | | | | | 0.000 | | | | | | | | |  |  |
| **2000** | **6** | **Treed Swamp / Coniferous Forest / Deciduous Forest / Thicket Swamp / Consistent Foraging Crop / StudyYear** | | | **-131** | | | | | | | | | **1.29** | | | | | | | | | | | | | **0.000** | | | | | | | | |  |  |
| ***Bombus* Species Proportional Abundance** | | | | | | | | | | | | | | | | |  | | | | | | | | | | |  | | | |  |  |  |  |  |  |
| 250 | 6 | Treed Swamp / Coniferous Forest / Mixed Forest / Deciduous Forest / Consistent Foraging Crop / StudyYear | | -179 | | | | | | | | 0.00 | | | | | | | | | | | 0.000 | | | | | | | | | | |  |  |  |  |
| 300 | 5 | Treed Swamp / Coniferous Forest / Deciduous Forest / Consistent Foraging Crop / StudyYear | | -186 | | | | | | | | -6.61 | | | | | | | | | | | 0.000 | | | | | | | | | | |  |  |  |  |
| 350 | 5 | Pasture / Thicket Swamp / Marsh / Consistent Foraging Crop / StudyYear | | -187 | | | | | | | | -7.24 | | | | | | | | | | | 0.000 | | | | | | | | | | |  |  |  |  |
| **400** | **6** | **Pasture / Marsh / Built Up Area Pervious / Consistent Foraging Crop / Tall Grass Woodland / StudyYear** | | **-214** | | | | | | | | **-34.30** | | | | | | | | | | | **0.829** | | | | | | | | | | |  |  |  |  |
| 450 | 6 | Pasture / Marsh / Built Up Area Pervious / Consistent Foraging Crop / Tall Grass Savanah / StudyYear | | -206 | | | | | | | | -27.21 | | | | | | | | | | | 0.024 | | | | | | | | | | |  |  |  |  |
| 500 | 5 | Pasture / Thicket Swamp / Marsh / Consistent Foraging Crop / StudyYear | | -210 | | | | | | | | -30.83 | | | | | | | | | | | 0.147 | | | | | | | | | | |  |  |  |  |
| **750** | **6** | **Deciduous Forest / Thicket Swamp / Marsh / Built Up Area Pervious / Consistent Foraging Crop / StudyYear** | | **-219** | | | | | | | | **0.00** | | | | | | | | | | | **0.000** | | | | | | | | | | |  |  |  |  |
| 1000 | 4 | Forest / Thicket Swamp / Abandoned Extraction Vegetated / StudyYear | | -204 | | | | | | | | 15.01 | | | | | | | | | | | 0.000 | | | | | | | | | | |  |  |  |  |
| 1250 | 6 | Forest / Thicket Swamp / Marsh / Hedge Row / Abandoned Extraction Vegetated / StudyYear | | -209 | | | | | | | | 9.79 | | | | | | | | | | | 0.000 | | | | | | | | | | |  |  |  |  |
| 1500 | 6 | Coniferous Forest / Built Up Area Pervious / Abandoned Extraction Vegetated / Tall Grass Woodland / Tall Grass Savanah / StudyYear | | -206 | | | | | | | | 0.00 | | | | | | | | | | | 0.000 | | | | | | | | | | |  |  |  |  |
| 1750 | 6 | Coniferous Forest / Thicket Swamp / Abandoned Extraction - Open / Tall Grass Woodland / Tall Grass Savanah / StudyYear | | -207 | | | | | | | | -1.09 | | | | | | | | | | | 0.000 | | | | | | | | | | |  |  |  |  |
| **2000** | **6** | **Coniferous Forest / Mixed Forest / Thicket Swamp / Abandoned Extraction - Open / Tall Grass Woodland / StudyYear** | | **-211** | | | | | | | | **-3.19** | | | | | | | | | | | **0.000** | | | | | | | | | | |  |  |  |  |
| **Cleptoparasite Proportional Abundance** | | | | | | | | | | | | | | | | |  | | | | | | | | | | |  | | | |  |  |  |  |  |  |
| 250 | 3 | Hedge Row / Semi-Natural (Shrub and Grassland) / Tall Grass Woodland | -126 | | | | | | 0.00 | | | | | | | | | | | | | 0.000 | | | | | | | | | |  |  |  |  |  |  |
| 300 | 1 | Tall Grass Woodland | -126 | | | | | | 0.39 | | | | | | | | | | | | | 0.000 | | | | | | | | | |  |  |  |  |  |  |
| 350 | 3 | Pasture / Consistent Foraging Crop / Tall Grass Woodland | -146 | | | | | | -19.98 | | | | | | | | | | | | | 0.002 | | | | | | | | | |  |  |  |  |  |  |
| 400 | 1 | Tall Grass Woodland | -140 | | | | | | -13.83 | | | | | | | | | | | | | 0.000 | | | | | | | | | |  |  |  |  |  |  |
| 450 | 3 | Coniferous Forest / Thicket Swamp / Tall Grass Woodland | -151 | | | | | | -25.16 | | | | | | | | | | | | | 0.027 | | | | | | | | | |  |  |  |  |  |  |
| **500** | **2** | **Deciduous Forest / Tall Grass Woodland** | **-159** | | | | | | **-32.36** | | | | | | | | | | | | | **0.971** | | | | | | | | | |  |  |  |  |  |  |
| 750 | 2 | Tall Grass Woodland / Tall Grass Savanah | -166 | | | | | | 0.00 | | | | | | | | | | | | | 0.000 | | | | | | | | | |  |  |  |  |  |  |
| 1000 | 2 | Tall Grass Woodland / Tall Grass Savanah | -166 | | | | | | 0.00 | | | | | | | | | | | | | 0.000 | | | | | | | | | |  |  |  |  |  |  |
| **1250** | 6 | **Treed Swamp / Coniferous Forest / Thicket Swamp / Hedge Row / Semi-Natural (Shrub and Grassland) / Tall Grass Savanah** | **-168** | | | | | | **-1.70** | | | | | | | | | | | | | **0.000** | | | | | | | | | |  |  |  |  |  |  |
| 1500 | 2 | Tall Grass Woodland / Tall Grass Savanah | -166 | | | | | | 0.00 | | | | | | | | | | | | | 0.000 | | | | | | | | | |  |  |  |  |  |  |
| 1750 | 2 | Tall Grass Woodland / Tall Grass Savanah | -166 | | | | | | 0.00 | | | | | | | | | | | | | 0.000 | | | | | | | | | |  |  |  |  |  |  |
| **2000** | **2** | **Tall Grass Woodland / Tall Grass Savanah** | **-166** | | | | | | **0.00** | | | | | | | | | | | | | **0.000** | | | | | | | | | |  |  |  |  |  |  |

**Table S8.** List of parameters of the best fit model at different spatial distances for species richness of each functional guild: solitary ground nesters, social ground nesters, cavity nesters, *Bombus* spp. and cleptoparasites. The number of habitat parameters and name of habitat parameters used in each model are listed. Coefficients are based on log-transformed data and in bold where 95% CIs do not include 0. Negative coefficients suggest habitat type has a negative impact on expected species richness and/or proportional abundance of functional guilds at each specific spatial scale. Greyed out habitat types were not model parameters in any candidate models for any functional guild. Numbers adjacent to land classes represent spatial distance categories (1= <500m; 2= 500-1500m; 3= >1500m).

|  | Solitary ground | | |  | Social ground | | |  | Cavity nesters | | |  | *Bombus* spp. | | |  | Cleptoparasites | | | |
| --- | --- | --- | --- | --- | --- | --- | --- | --- | --- | --- | --- | --- | --- | --- | --- | --- | --- | --- | --- | --- |
|  |  |  |  |  |  |  |  |  |  |  |  |  |  |  |  |  |  |  | |  |
| Land Classes | *β* | Lower CI | Upper CI |  | *β* | Lower CI | Upper CI |  | *β* | Lower CI | Upper CI |  | *β* | Lower CI | Upper CI |  | *β* | Lower CI | | Upper CI |
|  |  |  |  |  |  |  |  |  |  |  |  |  |  |  |  |  |  | |  |  |
| 1 Intercept |  |  |  |  | **9.7** | **6.2** | **9.8** |  | **15.5** | **7.4** | **9.8** |  |  |  |  |  | **15.4** | | **6.5** | **8.6** |
| 2 |  |  |  |  |  |  |  |  |  |  |  |  | **11.6** | **8.7** | **12.61** |  | **9.3** | | **4.3** | **6.9** |
| 3 | 6.7 | 10.6 |  |  | **9.5** | **7.9** | **12.5** |  |  |  |  |  | **15.4** | **8.7** | **11.5** |  |  | |  |  |
| 1 Aband Extract Veg |  |  |  |  |  |  |  |  |  |  |  |  |  |  |  |  |  | |  |  |
| 2 |  |  |  |  |  |  |  |  |  |  |  |  | **18.8** | **0.7** | **0.8** |  |  | |  |  |
| 3 |  |  |  |  |  |  |  |  |  |  |  |  |  |  |  |  |  | |  |  |
| 1 Aband Extract Open  2 |  |  |  |  |  |  |  |  |  |  |  |  |  |  |  |  |  | |  |  |
|  |  |  |  |  |  |  |  |  |  |  |  |  |  |  |  |  |  | |  |  |
| 3 |  |  |  |  |  |  |  |  | **9.8** | **0.3** | **0.4** |  |  |  |  |  |  | |  |  |
| 1 Built Up Pervious  2 |  |  |  |  |  |  |  |  |  |  |  |  |  |  |  |  |  | |  |  |
|  | 2.1 | 0.0 | 0.2 |  | **4.4** | **0.4** | **1.1** |  |  |  |  |  |  |  |  |  |  | |  |  |
| 3 |  |  |  |  |  |  |  |  |  |  |  |  |  |  |  |  | **4.8** | | **0.1** | **0.3** |
| 1 Coniferous Forest  2 |  |  |  |  |  |  |  |  |  |  |  |  | **-13.2** | **-0.5** | **-0.4** |  |  | |  |  |
|  |  |  |  |  |  |  |  |  |  |  |  |  |  |  |  |  | **6.8** | | **0.4** | **0.8** |
| 3 | **-5.4** | **-0.2** | **-0.1** |  | -2.3 | -0.3 | 0.0 |  | **-2.7** | **-0.4** | **-0.1** |  |  |  |  |  |  | |  |  |
| 1 Consis Forage Crop | -2.4 | -0.1 | 0.0 |  | **-6.3** | **-5.5** | **-2.7** |  | **-3.6** | **-1.1** | **-0.3** |  |  |  |  |  | **-5.7** | | **-2.6** | **-1.2** |
| 2 |  |  |  |  | 1.2 | -0.2 | 0.6 |  |  |  |  |  |  |  |  |  |  | |  |  |
| 3 |  |  |  |  |  |  |  |  |  |  |  |  |  |  |  |  |  | |  |  |
| 1 Deciduous Forest  2 | 1.3 | -0.1 | 0.3 |  |  |  |  |  |  |  |  |  |  |  |  |  | **7.4** | | **0.8** | **1.5** |
|  |  |  |  |  | **4.3** | **0.2** | **0.5** |  |  |  |  |  | **-4.8** | **-0.2** | **-0.1** |  |  | |  |  |
| 3 | -2.5 | -0.1 | 0.0 |  |  |  |  |  | **-4.4** | **-0.7** | **-0.3** |  | -6.5 | 0.0 | 0.0 |  |  | |  |  |
| 1 Forest  2 |  |  |  |  | **7.4** | **9.3** | **17.1** |  | **8.2** | **6.5** | **11.2** |  | **11.8** | **0.2** | **0.4** |  |  | |  |  |
|  | -1.9 | -0.2 | 0.0 |  |  |  |  |  |  |  |  |  |  |  |  |  | **4.7** | | **1.0** | **2.8** |
| 3 | **3.9** | **0.5** | **1.9** |  |  |  |  |  | **13.0** | **0.8** | **1.2** |  | **4.6** | **0.1** | **0.4** |  |  | |  |  |
| 1 Hedge Row |  |  |  |  |  |  |  |  |  |  |  |  |  |  |  |  |  | |  |  |
| 2 |  |  |  |  |  |  |  |  | -1.8 | -0.3 | 0.0 |  |  |  |  |  |  | |  |  |
| 3 |  |  |  |  |  |  |  |  |  |  |  |  |  |  |  |  |  | |  |  |
| 1 Marsh  2 |  |  |  |  |  |  |  |  |  |  |  |  | **13.8** | **0.5** | **0.7** |  |  | |  |  |
|  |  |  |  |  | **-3.9** | **-1.0** | **-0.3** |  |  |  |  |  |  |  |  |  |  | |  |  |
| 3 |  |  |  |  |  |  |  |  |  |  |  |  |  |  |  |  |  | |  |  |
| 1Mixed Forest  2 |  |  |  |  |  |  |  |  |  |  |  |  | 2.2 | 0.0 | 0.3 |  |  | |  |  |
|  |  |  |  |  |  |  |  |  |  |  |  |  |  |  |  |  |  | |  |  |
| 3 |  |  |  |  |  |  |  |  | 3.9 | 0.2 | 0.5 |  |  |  |  |  |  | |  |  |
| 1Pasture  2 |  |  |  |  | 5.8 | 0.6 | 1.2 |  |  |  |  |  |  |  |  |  |  | |  |  |
|  |  |  |  |  |  |  |  |  |  |  |  |  |  |  |  |  | -3.7 | | 0.0 | 0.0 |
| 3 |  |  |  |  |  |  |  |  |  |  |  |  | -3.2 | 0.0 | 0.0 |  | 1.4 | | -0.1 | 0.3 |
| 1Plantation  2 |  |  |  |  | **6.2** | **1.3** | **2.7** |  |  |  |  |  |  |  |  |  |  | |  |  |
|  |  |  |  |  |  |  |  |  | **-23.3** | **-0.5** | **-0.4** |  |  |  |  |  | -6.7 | | -0.5 | -0.3 |
| 3 | **4.5** | **0.1** | **0.1** |  |  |  |  |  |  |  |  |  |  |  |  |  |  | |  |  |
| 1Semi-Natural  2 |  |  |  |  |  |  |  |  | **6.8** | **0.4** | **0.7** |  |  |  |  |  | **-5.5** | | **-1.9** | **-0.8** |
|  |  |  |  |  |  |  |  |  |  |  |  |  | **6.1** | **0.1** | **0.2** |  |  | |  |  |
| 3 |  |  |  |  | **2.3** | **0.0** | **0.2** |  |  |  |  |  | **6.6** | **0.1** | **0.1** |  | **-3.4** | | **-0.4** | **-0.1** |
| 1Tallgrass Savannah  2 | **-2.8** | **-0.4** | **-0.1** |  |  |  |  |  |  |  |  |  |  |  |  |  |  | |  |  |
|  |  |  |  |  |  |  |  |  |  |  |  |  | **6.0** | **7.3** | **15.3** |  |  | |  |  |
| 3 | **3.9** | **5.8** | **19.5** |  |  |  |  |  | **15.1** | **0.5** | **0.7** |  | **9.0** | **3.3** | **5.4** |  | **-27.0** | | **-2.5** | **-2.1** |
| 1Tallgrass Wood  2 |  |  |  |  |  |  |  |  | **-13.6** | **-0.3** | **-0.2** |  |  |  |  |  | **47.3** | | **0.8** | **0.8** |
|  |  |  |  |  |  |  |  |  |  |  |  |  |  |  |  |  |  | |  |  |
| 3 |  |  |  |  |  |  |  |  |  |  |  |  |  |  |  |  | **26.0** | | **2.7** | **3.2** |
| 1Thicket Swamp  2 | **-7.3** | **-0.2** | **-0.1** |  |  |  |  |  |  |  |  |  |  |  |  |  |  | |  |  |
|  | **-6.4** | **-0.4** | **-0.2** |  |  |  |  |  | **-6.8** | **-0.6** | **-0.3** |  | **-4.9** | **-0.4** | **-0.1** |  |  | |  |  |
| 3 |  |  |  |  | **-5.0** | **-0.3** | **-0.1** |  |  |  |  |  |  |  |  |  |  | |  |  |
| 1Treed Sand  2 |  |  |  |  |  |  |  |  |  |  |  |  |  |  |  |  |  | |  |  |
|  |  |  |  |  |  |  |  |  |  |  |  |  |  |  |  |  |  | |  |  |
| 3 |  |  |  |  |  |  |  |  |  |  |  |  |  |  |  |  |  | |  |  |
| 1Treed Swamp  2 | 4.1 | 0.5 |  |  | **9.2** | **1.1** | **1.9** |  | **13.3** | **1.0** | **1.4** |  | **10.7** | **3.3** | **5.1** |  |  | |  |  |
|  | 4.9 | 0.6 | 1.5 |  | **3.7** | **0.9** | **3.3** |  | **9.6** | **1.0** | **1.6** |  |  |  |  |  | -2.4 | | -0.1 | 0.0 |
| 3 |  |  |  |  | **3.9** | **0.0** | **0.1** |  |  |  |  |  |  |  |  |  |  | |  |  |
| 1StudyYear-2002 | -1.3 | -0.7 | 0.2 |  | **-7.2** | **-48.0** | **-25.5** |  | **-12.0** | **-66.7** | **-46.4** |  | **-12.5** | **-4.5** | **-3.2** |  | **48.1** | | **22.4** | **24.5** |
| 2 | -0.7 | -0.4 | 0.2 |  | **-2.5** | **-1.8** | **-0.1** |  | **-7.9** | **-0.9** | **-0.5** |  | **-18.1** | **-6.0** | **-4.7** |  | 1.0 | | -3.0 | 7.9 |
| 3 | **17.4** | **29.7** | **38.1** |  | 0.2 | -7.8 | 9.6 |  |  |  |  |  | **-5.2** | **-4.6** | **-1.9** |  | **19.7** | | **0.4** | **0.5** |
| 1StudyYear-2003 | **16.0** | **0.2** | **0.3** |  | **21.9** | **16.2** | **19.8** |  | **55.2** | **10.9** | **11.8** |  | **-12.5** | **-4.5** | **-3.2** |  | **19.8** | | **8.3** | **10.4** |
| 2 | **15.7** | **0.3** | **0.3** |  | **12.3** | **0.4** | **0.6** |  | **22.6** | **0.2** | **0.2** |  | **7.6** | **3.3** | **6.0** |  | **2.2** | | **0.0** | **0.8** |
| 3 | **3.7** | **3.6** | **13.7** |  | **16.7** | **15.6** | **20.2** |  |  |  |  |  | **-3.6** | **-3.3** | **-0.8** |  | **13.8** | | **0.1** | **0.2** |
| 1StudyYear-2006 | **16.0** | **0.2** | **0.3** |  | **21.9** | **16.2** | **19.8** |  | **55.2** | **10.9** | **11.8** |  | **-12.5** | **-4.5** | **-3.2** |  | **19.8** | | **8.3** | **10.4** |
| 2 | **15.7** | **0.3** | **0.3** |  | **12.3** | **0.4** | **0.6** |  | **22.6** | **0.2** | **0.2** |  | **7.6** | **3.3** | **6.0** |  | **2.2** | | **0.0** | **0.8** |
| 3 | **3.7** | **3.6** | **13.7** |  | **16.7** | **15.6** | **20.2** |  |  |  |  |  | **-3.6** | **-3.3** | **-0.8** |  | **13.8** | | **0.1** | **0.2** |
| 1StudyYear-2009 | -0.1 | -0.5 | 0.4 |  | **-6.0** | **-42.0** | **-19.5** |  | -11.2 | -62.7 | -42.4 |  | **-17.2** | **-1.6** | **-1.3** |  | **52.2** | | **24.4** | **26.5** |
| 2 | 1.2 | -0.1 | 0.4 |  | -1.9 | -1.6 | 0.1 |  | -6.5 | -0.7 | -0.4 |  | **-18.1** | **-6.0** | **-4.7** |  | 1.0 | | -3.0 | 7.9 |
| 3 | **25.1** | **44.7** | **53.1** |  | 1.7 | -1.8 | 15.6 |  |  |  |  |  | **-5.2** | **-4.6** | **-1.9** |  | **21.5** | | **0.5** | **0.6** |
| 1StudyYear-2010 | -1.0 | -0.2 | 0.1 |  | **2.7** | **0.6** | **6.2** |  | -2.6 | -2.6 | -0.3 |  | **-31.9** | **-1.2** | **-1.0** |  | -2.0 | | -4.2 | 0.1 |
| 2 | -1.5 | -0.1 | 0.0 |  | **9.3** | **0.7** | **1.1** |  | -10.4 | -0.7 | -0.4 |  | **-11.3** | **-8.7** | **-5.9** |  | **-7.0** | | **-3.5** | **-1.9** |
| 3 | -1.7 | -6.2 | 0.7 |  | 0.8 | -2.3 | 4.8 |  |  |  |  |  | **-9.6** | **-8.2** | **-5.2** |  | -1.1 | | -0.2 | 0.1 |
| 1StudyYear-2014  2 |  |  |  |  |  |  |  |  |  |  |  |  |  |  |  |  |  | |  |  |
| 3 |  |  |  |  |  |  |  |  |  |  |  |  |  |  |  |  |  | |  |  |

**Table S9.** List of parameters of the best fit model at different spatial distances for species abundance of each functional guild: solitary ground nesters, social ground nesters, cavity nesters, *Bombus* spp. and cleptoparasites. The number of habitat parameters and name of habitat parameters used in each model are listed. Coefficients are based on log-transformed data and in bold where 95% CIs do not include 0. Negative coefficients suggest habitat type has a negative impact on expect species richness and/or proportional abundance of functional guilds at each specific spatial scale. Greyed out habitat types were not model parameters in any candidate models for any functional guild. Numbers adjacent to land classes represent spatial distance categories (1= <500m; 2= 500-1500m; 3= >1500m).

|  | Solitary ground | | |  | Social ground | | |  | Cavity nesters | | |  | *Bombus* spp. | | |  | Cleptoparasites | | | |
| --- | --- | --- | --- | --- | --- | --- | --- | --- | --- | --- | --- | --- | --- | --- | --- | --- | --- | --- | --- | --- |
|  |  |  |  |  |  |  |  |  |  |  |  |  |  |  |  |  |  |  | |  |
| Land Classes | *β* | Lower CI | Upper CI |  | *β* | Lower CI | Upper CI |  | *β* | Lower CI | Upper CI |  | *β* | Lower CI | Upper CI |  | *β* | Lower CI | | Upper CI |
|  |  |  |  |  |  |  |  |  |  |  |  |  |  |  |  |  |  | |  |  |
| 1 Intercept |  |  |  |  |  |  |  |  |  |  |  |  |  |  |  |  |  | |  |  |
| 2 | **3.0** | **0.1** | **0.9** |  | **7.5** | **0.2** | **0.4** |  |  |  |  |  |  |  |  |  |  | |  |  |
| 3 |  |  |  |  |  |  |  |  | **20.7** | **0.4** | **0.5** |  |  |  |  |  |  | |  |  |
| 1Aband Extract Veg |  |  |  |  |  |  |  |  |  |  |  |  |  |  |  |  |  | |  |  |
| 2 |  |  |  |  |  |  |  |  | **-4.4** | **-0.4** | **-0.1** |  |  |  |  |  |  | |  |  |
| 3 | **-4.4** | **-1.2** | **-0.4** |  |  |  |  |  |  |  |  |  |  |  |  |  |  | |  |  |
| 1 Aband Extract Open  2 |  |  |  |  |  |  |  |  |  |  |  |  |  |  |  |  |  | |  |  |
|  |  |  |  |  |  |  |  |  |  |  |  |  |  |  |  |  |  | |  |  |
| 3 |  |  |  |  |  |  |  |  |  |  |  |  | **-47.7** | **-0.6** | **-0.5** |  |  | |  |  |
| 1 Built Up Pervious  2 |  |  |  |  |  |  |  |  |  |  |  | **-1201.3** | | **-1.1** | **-1.1** |  |  | |  |  |
|  |  |  |  |  |  |  |  |  |  |  |  |  | **-2.3** | **-0.5** | **-0.01** |  |  | |  |  |
| 3 | **2.6** | **0.1** | **1.5** |  |  |  |  |  |  |  |  |  |  |  |  |  |  | |  |  |
| 1 Coniferous Forest  2 | **-4.9** | **-0.9** | **-0.4** |  |  |  |  |  | **-6.0** | **-1.2** | **-0.6** |  |  |  |  |  |  | |  |  |
|  | **3.8** | **0.01** | **0.05** |  | **-6.3** | **-0.05** | **-0.02** |  | **-3.6** | **-0.6** | **-0.2** |  |  |  |  |  | **9.5** | | **1.0** | **1.6** |
| 3 |  |  |  |  |  |  |  |  | **-6.5** | **-0.004** | **-0.002** |  | **2.4** | **0.01** | **0.2** |  |  | |  |  |
| 1 Consist Forage Crop |  |  |  |  |  |  |  |  | **-2.6** | **-0.5** | **-0.04** |  | **12.8** | **0.1** | **0.2** |  |  | |  |  |
| 2 |  |  |  |  |  |  |  |  |  |  |  |  | **2.2** | **0.005** | **0.2** |  |  | |  |  |
| 3 |  |  |  |  |  |  |  |  | **-2.03** | **-0.01** | **0.00** |  |  |  |  |  |  | |  |  |
| 1 Deciduous Forest  2 |  |  |  |  |  |  |  |  |  |  |  |  |  |  |  |  | **-3.2** | | **-0.5** | **-0.1** |
|  | -1.8 | -0.03 | 0.002 |  |  |  |  |  |  |  |  |  | **-2.3** | **-0.4** | **-0.009** |  |  | |  |  |
| 3 |  |  |  |  |  |  |  | **-8.573** | | **-0.002** | **-0.001** |  |  |  |  |  |  | |  |  |
| 1 Forest  2 |  |  |  |  |  |  |  |  |  |  |  |  |  |  |  |  |  | |  |  |
|  |  |  |  |  |  |  |  |  | **6.7** | **0.4** | **0.7** |  |  |  |  |  |  | |  |  |
| 3 |  |  |  |  |  |  |  |  |  |  |  |  |  |  |  |  |  | |  |  |
| 1 Hedge Row |  |  |  |  |  |  |  |  |  |  |  |  |  |  |  |  |  | |  |  |
| 2 | 1.8 | -0.002 | 0.03 |  | -2.1 | -0.032 | 0.000 |  |  |  |  |  |  |  |  |  | **2.5** | | **0.1** | **1.0** |
| 3 |  |  |  |  |  |  |  |  |  |  |  |  |  |  |  |  |  | |  |  |
| 1 Marsh  2 | **-2.5** | **-0.6** | **-0.04** |  |  |  |  |  | **-5.9** | **-0.4** | **-0.2** |  | **10.10** | **0.08** | **01** |  |  | |  |  |
|  |  |  |  |  |  |  |  |  | **-8.4** | **-0.5** | **-0.3** |  | **9.0** | **0.5** | **0.8** |  |  | |  |  |
| 3 |  |  |  |  |  |  |  |  |  |  |  |  |  |  |  |  |  | |  |  |
| 1Mixed Forest  2 | **7.4** | **0.8** | **1.4** |  | **-3.0** | **-0.4** | **-0.06** |  |  |  |  |  |  |  |  |  |  | |  |  |
|  |  |  |  |  |  |  |  |  |  |  |  |  |  |  |  |  |  | |  |  |
| 3 |  |  |  |  |  |  |  |  |  |  |  |  | **-5.5** | **-0.4** | **-0.2** |  |  | |  |  |
| 1Pasture  2 |  |  |  |  | **2.9** | **0.1** | **0.8** |  |  |  |  |  | **-6.6** | **-0.2** | **-0.1** |  |  | |  |  |
|  |  |  |  |  |  |  |  |  | **-3.3** | **-0.8** | **-0.2** |  |  |  |  |  |  | |  |  |
| 3 |  |  |  |  |  |  |  |  |  |  |  |  |  |  |  |  |  | |  |  |
| 1 Plantation  2 |  |  |  |  | **2.9** | **0.04** | **0.3** |  |  |  |  |  |  |  |  |  |  | |  |  |
|  |  |  |  |  | **4.1** | **0.007** | **0.02** |  |  |  |  |  |  |  |  |  |  | |  |  |
| 3 |  |  |  |  |  |  |  |  |  |  |  |  |  |  |  |  |  | |  |  |
| 1 Semi-Natural  2 |  |  |  |  |  |  |  |  | **8.8** | **0.2** | **0.4** |  |  |  |  |  |  | |  |  |
|  |  |  |  |  | 1.9 | -0.003 | 0.05 |  |  |  |  |  |  |  |  |  | **-4.2** | | **-0.5** | **-0.2** |
| 3 |  |  |  |  | **2.2** | **0.02** | **0.6** |  |  |  |  |  |  |  |  |  |  | |  |  |
| 1 Tallgrass Savannah  2 | **8.8** | **0.9** | **1.4** |  | -2.1 | -1.1 | 0.03 |  |  |  |  |  |  |  |  |  |  | |  |  |
|  | **2.6** | **0.2** | **1.9** |  |  |  |  |  | **-10.7** | **-0.5** | **-0.3** |  |  |  |  |  | **8.1** | | **0.3** | **0.4** |
| 3 | **-4.2** | **-3.643** | **-1.194** |  |  |  |  |  |  |  |  |  |  |  |  | **-29.378** | | | **-3.846** | **-3.339** |
| 1 Tallgrass Wood  2 |  |  |  |  |  |  |  |  |  |  |  |  | **649.9** | **5.1** | **5.2** |  | **18.9** | | **0.8** | **1.0** |
| 3 |  |  |  |  |  |  |  |  |  |  |  |  |  |  |  |  |  | |  |  |
|  | **4.4** | **1.8** | **5.2** |  |  |  |  |  |  |  |  |  | **-7.5** | **-0.2** | **-0.1** |  | **26.8** | | **3.7** | **4.3** |
| 1 Thicket Swamp  2 | **3.4** | **0.1** | **0.5** |  |  |  |  |  |  |  |  |  |  |  |  |  |  | |  |  |
|  |  |  |  |  |  |  |  |  |  |  |  |  | **-5.0** | **-0.3** | **-0.1** |  | **3.6** | | **0.2** | **0.6** |
| 3 | **-4.7** | **-0.4** | **-0.2** |  | **8.7** | **0.3** | **0.5** |  | **-4.1** | **-0.006** | **-0.002** |  | **6.7** | **0.08** | **0.16** |  |  | |  |  |
| 1 Treed Sand  2 |  |  |  |  |  |  |  |  |  |  |  |  |  |  |  |  |  | |  |  |
| 3 |  |  |  |  |  |  |  |  |  |  |  |  |  |  |  |  |  | |  |  |
| 1 Treed Swamp  2 |  |  |  |  | 1.8 | -0.9 | 8.6 |  | 2.1 | -0.03 | 2.9 |  |  |  |  |  |  | |  |  |
|  | **-3.9** | **-0.008** | **-0.002** |  | **4.1** | **0.003** | **0.009** |  |  |  |  |  |  |  |  |  | **-6.5** | | **-1.6** | **-0.8** |
| 3 |  |  |  |  |  |  |  |  | **1.984** | **0.000** | **0.001** |  |  |  |  |  |  | |  |  |
| 1 StudyYear2002 | **-5.7** | **-0.9** | **-0.4** |  | -1.4 | -5.7 | 1.2 |  | **-2.9** | **-2.5** | **-0.3** | **-1786.9** | | **-1.2** | **-1.2** |  |  | |  |  |
| 2 | 1.5 | -0.1 | 0.8 |  | -0.7 | -0.6 | 0.3 |  |  |  |  |  | **-12.2** | **-0.5** | **-0.4** |  |  | |  |  |
| 3 | -1.5 | -0.3 | 0.05 |  | **22.3** | **0.4** | **0.5** |  | -0.4 | -0.1 | 0.1 |  | **-41.6** | **-0.8** | **-0.7** |  |  | |  |  |
| 1 StudyYear-2003 | -0.08 | -0.2 | 0.2 |  | -1.8 | -6.2 | 0.7 |  | **19.9** | **0.4** | **0.5** | **-1852.7** | | **-1.3** | **-1.2** |  |  | |  |  |
| 2 | 1.0 | -0.2 | 0.5 |  | **3.3** | **0.04** | **0.2** |  |  |  |  |  | **-44.4** | **-0.5** | **-0.4** |  |  | |  |  |
| 3 | **-6.1** | **-0.7** | **-0.3** |  | **4.9** | **0.06** | **0.2** |  | **27.6** | **0.3** | **0.4** | **-231.4** | | **-0.9** | **-0.9** |  |  | |  |  |
| 1 StudyYear-2006 | -1.7 | -0.41 | 0.055 |  | 0.2 | -0.166 | 0.200 |  | **-2.5** | **-2.28** | **-0.14** | **-1916.7** | | **-1.33** | **-1.3** |  |  | |  |  |
| 2 | **3.1** | **0.19** | **1.07** |  | **-2.8** | **-1.036** | **-0.142** |  |  |  |  | **-14.607** | | **-0.61** | **-0.46** |  |  | |  |  |
| 3 | **4.89** | **0.19** | **0.49** |  | -0.1 | -0.05 | 0.042 |  | **1.8** | **-0.022** | **0.22** |  | **-46.2** | **-0.92** | **-0.836** |  |  | |  |  |
| 1 StudyYear-2009 | **2.8** | **0.097** | **0.76** |  | **-2.9** | **-0.61** | **-0.077** |  | **-3.8** | **-0.36** | **-0.10** | **-102.9** | | **-2.86** | **-2.74** |  |  | |  |  |
| 2 | -1.3 | -0.59 | 0.14 |  | **3.8** | **0.081** | **0.29** |  |  |  |  |  | **-10.9** | **-1.59** | **-1.07** |  |  | |  |  |
| 3 | 0.2 | -0.29 | 0.33 |  | **3.2** | **0.10** | **0.49** |  | **-3.5** | **-0.13** | **-0.032** |  | **-77.2** | **-2.19** | **-2.07** |  |  | |  |  |
| 1 StudyYear-2010 | 1.5 | -0.092 | 0.47 |  |  |  |  |  | **10.3** | **0.50** | **0.77** | **-864.9** | | **-6.61** | **-6.58** |  |  | |  |  |
| 2 | -1.3 | -0.49 | 0.12 |  | -1.8 | -0.27 | 0.024 |  |  |  |  |  | **-28.9** | **-0.79** | **-0.68** |  |  | |  |  |
| 3 | **-3.0** | **-1.45** | **-0.25** |  | **-2.2** | **-0.56** | **-0.01** |  | **4.7** | **0.040** | **0.11** |  | **-53.0** | **-1.34** | **-1.24** |  |  | |  |  |
| 1 StudyYear-2014 |  |  |  |  |  |  |  |  |  |  |  |  |  |  |  |  |  | |  |  |
| 2 |  |  |  |  |  |  |  |  |  |  |  |  |  |  |  |  |  | |  |  |
| 3 |  |  |  |  |  |  |  |  |  |  |  |  |  |  |  |  |  | |  |  |

**References**

63 Sharkey, J. K., Pindar, A. & Raine, N.E. First Canadian record of the specialist Hibiscus Bee, *Pthilothrix bombiformis* (Cresson) (Hymenoptera: Apidae) *Journal of the Entomological Society of Ontario* **151**, 41-48 (2020).

64 Petchey, O. L. & Gaston, K. J. Functional diversity: back to basics and looking forward. *Ecology Letters* **9**, 741-758, doi:10.1111/j.1461-0248.2006.00924.x (2006).

65 Cadotte, M. W., Carscadden, K. & Mirotchnick, N. Beyond species: functional diversity and the maintenance of ecological processes and services. *Journal of Applied Ecology* **48**, 1079-1087, doi:10.1111/j.1365-2664.2011.02048.x (2011).

66 Hoehn, P., Tscharntke, T., Tylianakis, J. M. & Steffan-Dewenter, I. Functional group diversity of bee pollinators increases crop yield. *Proceedings of the Royal Society B: Biological Sciences* **275**, 2283-2291, doi:10.1098/rspb.2008.0405 (2008).

67 Neame, L. A., Griswold, T. & Elle, E. Pollinator nesting guilds respond differently to urban habitat fragmentation in an oak‐savannah ecosystem. *Insect Conservation and Diversity* **6**, 57-66, doi:10.1111/j.1752-4598.2012.00187.x (2013).

68 Williams, N. M. *et al.* Ecological and life-history traits predict bee species responses to environmental disturbances. *Biological Conservation* **143**, 2280-2291, doi:10.1016/j.biocon.2010.03.024 (2010).

69 Sheffield, C. S., Pindar, A., Packer, L. & Kevan, P. G. The potential of cleptoparasitic bees as indicator taxa for assessing bee communities. *Apidologie* **44**, 501-510, doi:10.1007/s13592-013-0200-2 (2013).

70 Gibbs, J. Revision of the metallic species of *Lasioglossum* (*Dialictus*) in Canada (Hymenoptera, Halictidae, Halictini). *Zootaxa* **3073**, 1-216, doi:10.11646/zootaxa.3073.1.1 (2011).

71 Cane, J. H., Griswold, T. L. & Parker, F. D. Substrates and materials used for nesting by North American *Osmia* bees (Hymenoptera: Apiformes: Megachilidae). *Annals of the Entomological Society of America* **100**, 350-358, doi:10.1603/0013-8746(2007)100[350:SAMUFN]2.0.CO;2 (2007).

72 Grixti, J. C. & Packer, L. Changes in the bee fauna (Hymenoptera: Apoidea) of an old field site in southern Ontario, revisited after 34 years. *Canadian Entomologist* **138**, 147-164, doi:10.4039/n05-034 (2006).

73 Richards, M. H. *et al.* Bee diversity in naturalizing patches of Carolinian grasslands in southern Ontario, Canada. *Canadian Entomologist* **143**, 279-299, doi:10.4039/N11-010 (2011).

74 Colla, S., Willis, E. & Packer, L. Can green roofs provide habitat for urban bees (Hymenoptera: Apidae)? *Cities and the Environment* **2**, 1-12 (2009).

75 Taylor, A. N. & Catling, P. M. Bees and butterflies in burned and unburned alvar woodland: evidence for the importance of postfire succession to insect pollinator diversity in an imperiled ecosystem. *The Canadian Field-Naturalist* **125**, 297-306, doi:10.22621/cfn.v125i4.1258 (2011).

76 Pindar, A. *The effect of fire disturbance on bee community composition in oak savannah habitat in southern Ontario, Canada.* PhD thesis, York University, (2014).

77 James, J. *Native bee diversity in conventional and organic hedgerows in Eastern*

*Ontario*. MSc thesis, University of Ottawa (2014).

78 Andrachuk, H. *The quality of citizen scientists’ bee observations: an evaluation of PollinatorWatch at Royal Botanical Gardens and the rare Charitable Research Reserve*. MSc thesis, University of Waterloo (2011).

79 Hogg, A. & Jones, C. D. A landscape-scale assessment of pollinator habitat in southern Ontario. 38 (Peterborough, Ontario, 2018).

80 Lee, H. Southern Ontario Ecological Land Classification. 35 (London, Ontario, 2008).

81 XLSTAT Statistical and data solutions software (Boston, USA, 2020).
